# Human plasma protein *N*-glycosylation

**DOI:** 10.1007/s10719-015-9626-2

**Published:** 2015-11-10

**Authors:** Florent Clerc, Karli R. Reiding, Bas C. Jansen, Guinevere S. M. Kammeijer, Albert Bondt, Manfred Wuhrer

**Affiliations:** Center for Proteomics and Metabolomics, Leiden University Medical Center, P.O. Box 9600, 2300 RC Leiden, The Netherlands; Department of Rheumatology, Leiden University Medical Center, Leiden, The Netherlands; Division of BioAnalytical Chemistry, VU University Amsterdam, Amsterdam, The Netherlands

**Keywords:** *N*-glycosylation, Plasma, Serum, Glycoproteins, Glycoproteomics, Immunoglobulins

## Abstract

Glycosylation is the most abundant and complex protein modification, and can have a profound structural and functional effect on the conjugate. The oligosaccharide fraction is recognized to be involved in multiple biological processes, and to affect proteins physical properties, and has consequentially been labeled a critical quality attribute of biopharmaceuticals. Additionally, due to recent advances in analytical methods and analysis software, glycosylation is targeted in the search for disease biomarkers for early diagnosis and patient stratification. Biofluids such as saliva, serum or plasma are of great use in this regard, as they are easily accessible and can provide relevant glycosylation information. Thus, as the assessment of protein glycosylation is becoming a major element in clinical and biopharmaceutical research, this review aims to convey the current state of knowledge on the *N*-glycosylation of the major plasma glycoproteins alpha-1-acid glycoprotein, alpha-1-antitrypsin, alpha-1B-glycoprotein, alpha-2-HS-glycoprotein, alpha-2-macroglobulin, antithrombin-III, apolipoprotein B-100, apolipoprotein D, apolipoprotein F, beta-2-glycoprotein 1, ceruloplasmin, fibrinogen, immunoglobulin (Ig) A, IgG, IgM, haptoglobin, hemopexin, histidine-rich glycoprotein, kininogen-1, serotransferrin, vitronectin, and zinc-alpha-2-glycoprotein. In addition, the less abundant immunoglobulins D and E are included because of their major relevance in immunology and biopharmaceutical research. Where available, the glycosylation is described in a site-specific manner. In the discussion, we put the glycosylation of individual proteins into perspective and speculate how the individual proteins may contribute to a total plasma *N*-glycosylation profile determined at the released glycan level.

**Electronic supplementary material:**

The online version of this article (doi:10.1007/s10719-015-9626-2) contains supplementary material, which is available to authorized users.

## Introduction

Protein glycosylation is recognized to be involved in a multitude of biological processes such as receptor interaction, immune response, protein secretion and transport [1–6]. In addition, glycosylation affects protein properties such as solubility, stability and folding [7–10]. A given protein can have multiple sites of glycosylation, and its glycoforms can differ by site occupancy (macroheterogeneity) and occupying glycan structures (microheterogeneity) [11–13]. The biosynthetic pathways leading up to this variety of glycans depend on multiple parameters and can be influenced by many factors including genetic regulation, the availability of nucleotide sugars, the time spent in the endoplasmic reticulum and Golgi apparatus, as well as the accessibility of a particular glycosylation site [10, 14–17].

Protein glycosylation can differ between persons, but is remarkably stable per individual [18]. It is only when the homeostasis of a person changes, by lifestyle or pathological conditions, that the glycosylation will change notably [19]. Large studies comprising thousands of individuals have identified glycosylation to correlate with age, sex and lifestyle [14, 20, 21]. Examples of such changes are the increase in bisection and decrease of galactosylation and sialylation of IgG with age [19, 20, 22–24].

At the same time, specific studies based on smaller sample sets have revealed changes of glycosylation in various diseases, inflammatory states, congenital disorders of glycosylation (CDGs), but also throughout pregnancy where an increase in galactosylation and sialylation, as well as a decrease in bisection was reported [25–28]. In addition, specific glycoforms can be targeted by viruses or bacteria or serve as a pro- or anti-inflammatory signal [29–33]. All of this opens up the possibility to use glycans as an early biomarker for disease or to assist personalized medicine by patient stratification [34, 35].

Recent advances in chromatographic separation, mass spectrometry, robotization and automated data processing allow the rapid analysis of glycosylation, and facilitate the development of novel biomarkers [36–39]. While the glycosylation analysis of an easily obtainable biofluid like plasma can be of considerable interest to a clinical situation, the interpretation of data may be complicated when analyzing a complex protein mixture. For example, when analyzing total plasma *N*-glycosylation (TPNG) of a clinical cohort at the released glycan level, it is not directly apparent whether an observed change originates from a change in relative protein abundance, in the relative glycoforms of a specific protein, or whether it reflects a general regulatory effect influencing the glycosylation of many different glycoproteins. We expect that a better understanding of the glycosylation of individual proteins of human plasma will help to put total plasma *N*-glycomic changes into perspective.

As the previous review on plasma protein *N*-glycosylation originates from 2008 [40], we here strive to convey the current state of knowledge on the subject, including a larger number of proteins. The proteins described in this review were selected based on their plasma levels, additionally including the immunoglobulin family due to its major clinical and biopharmaceutical interest. The 24 glycoproteins covered in this review account for approximately 30 mg/mL of the 70–75 mg/mL of the total plasma protein concentration, thus representing most of the human TPNG (albumin is present at levels of 40 mg/mL but is not glycosylated) [41–43].

**Fig. 1:**
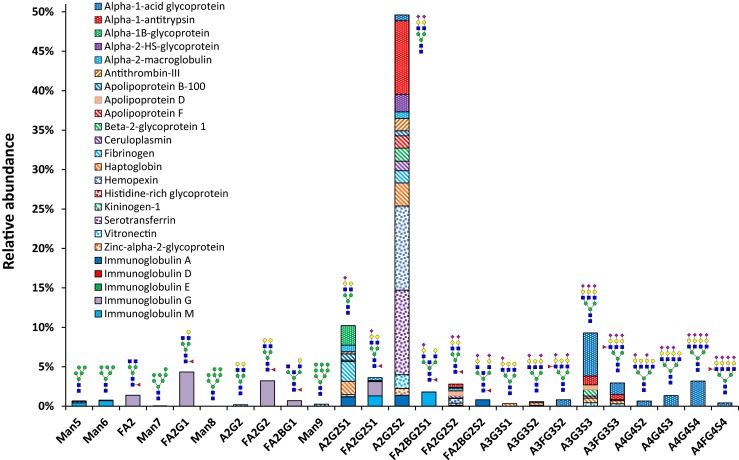
Schematic representation of the relative protein contribution to each specific glycan composition. To obtain these numbers, the contribution of a glycan composition to the total glycan pool of a given protein was multiplied by the abundance of that protein as well as the number of glycosylation sites confirmed to be occupied. Protein concentrations were taken from large studies when available and a mean value was calculated from the reported ranges otherwise. The molecular mass used is as reported by SDS-PAGE for the glycoproteins or calculated from the phenotype distribution for haptoglobin. The general Oxford notation was used for naming the glycan structures. For details on the calculation see Supplementary Table 1

Furthermore, we tried to limit our review to human plasma and serum but we also reported findings coming from other biological fluids when information complementary information could be added. The *N*-glycosylation of the proteins is reported both on a general level and, where available, with site specific information about glycan composition, glycan structure and occupancy. The information is condensed in Table 1, and a schematic representation of the relative protein contribution to each specific glycan composition is reported in Fig. 1.

**Table 1:** Overview on plasma protein glycosylation

Glycoprotein	Uniprot number	Function	Plasma concentration range (mg/mL)	Avg. plasma concentration (mg/mL)	*N*-glycosylation sites	Site occupancy (%)	Glycan species	Changes under disease or inflammation	Changes with age	Glycosylation references (numbered)
Alpha-1-acid glycoprotein	P02763; P19652	Transport of lipophilic compounds	0.36–1.46	0.77	Overall		A3G3S3 (33.5 %); A4G4S4 (19.5 %); A3FG3S3 (9 %); A4G4S3 (8.2 %); A3G3S2 (5 %); A2G2S2 (4.5 %); A4G4S2 (4 %); A4FG4S4 (2.5 %)	Conc↑, F↑, S↑	Conc↑ for females	[44–51]
					Asn33		A3G3S3 (60 %); A3FG3S3 (20 %); A3G3S2 (12.5 %)			
					Asn56		A3G3S3 (55 %); A2G2S2 (22.5 %); A3FG3S3 (12.5 %); A3G3S2 (10 %);			
					Asn72		A4G4S4 (30 %); A4G4S3 (15 %); A3G3S3 (15 %); A4G4S2 (10 %); A4FG4S3 (5 %); A4FG4S4 (5 %)			
					Asn93		A4G4S4 (22.5 %); A4G4S3 (20 %); A3G3S3 (17.5 %); A4FG4S4 (7.5 %); A4FG3S3 (7.5 %); A3FG3S3 (7.5 %); A4G4S2 (7.5 %)			
					Asn103		A4G4S4 (45 %); A3G3S3 (20 %); A4G4S2 (10 %); A4G4S3 (7.5 %); A3FG3S3 (5 %)			
Alpha-1-antitripsin	P01009	Serine protease inhibitor	1.1	1.1	Overall		A2G2S2 (81 %); A3G3S3 (9.8 %); A3FG3S3 (5.6 %); FA2G2S2 (3.6 %)	Conc↑, complex	Glycosylation↓ except A2G1 and A3FG2S2↑	[52–57]
					Asn70		A2G2S2 (91.3 %); FA2G2S2 (8.6 %)			
					Asn107		A2G2S2 (52.5 %); A3G3S3 (29.5 %); A3FG3S3 (16.7 %); FA2G2S2 (1.5 %)			
					Asn271		A2G2S2 (99.3 %); FA2G2S2 (0.7 %)			
Alpha-1B-glycoprotein	P04217	Uncertain, likely inflammation	0.22	0.22	Overall		A2G2S1 (100 %)			[58–62]
					Asn44					
					Asn179					
					Asn363		A2G2S1 (100 %)			
					Asn371					
Alpha-2-HS-glycoprotein	P02765	Phosphate and calcium scavenger, metalloprotease protection	0.3–0.6	0.45	Overall		A2G2S2 (96 %); FA2G2S2 (4 %)	Conc↑, (F)A3G3S3↑, A2G2S2↓		[63–68]
					Asn156			Glycosylation ↑↓		
					Asn176					
Alpha-2-macrogobulin	P01023	Protease scavenger	1–2	1.2	Overall		A2G2S2 (35 %); A2G2S1 (35 %); FA2G2S2 (15 %); FA2G2S1 (15 %); M5-7 (8 %)	General ↑, Man↑, G↑		[59, 60, 67, 69–74]
					Asn55		A2G2S2; FA2G2S2			
					Asn70		A2G2S2			
					Asn247		A2G2S2			
					Asn396		A2G2S2			
					Asn410		A2G2S2			
					Asn869		Man5-7			
					Asn991		A2G2S2			
					Asn1424		A2G2S2; FA2G2S2			
Antithrombin-III	P01008	Serine protease inhibitor, coagulation	0.15	0.15	Overall		A2G2S2 (85 %); A2G2S1 (15 %)	Conc↓ (thrombosis), FA↑		[75–86]
					Asn128		A2G2S2; A2G2S1			
					Asn167		A2G2S2; A2G2S1; A3G3S3			
					Asn187		A2G2S2; A2G2S1; FA2G2S2; A3G3S3			
					Asn224		A2G2S2; A2G2S1; A3G3S2			
Apolipoprotein B-100	P04114	Cholesterol transport (LDL)	0.5	0.5	Overall		A2G2S1 (29.2 %); A2G2S2 (23.6 %); Man9 (8.6 %); A2G2 (7.2 %); Man5 (6.9 %)			[11, 87–90]
					Asn34	0 %	---			
					Asn185		Man5-9			
					Asn983		A2G2S1; A2G2S2			
					Asn1368		Man5-9			
					Asn1377		Man5-9			
					Asn1523		Man5-9; Hy; A1G1S1; A2G2S1; A2G2S2			
					Asn2239		A2G2S1; A2G2S2			
					Asn2560	0 %	---			
					Asn2779		A2G2S1; A2G2S2			
					Asn2982		A2G2S1; A2G2S2			
					Asn3101		A2G2S1; A2G2S2			
					Asn3224		A2G2S1; A2G2S2			
					Asn3336		Man5-9			
					Asn3358		Man5-9			
					Asn3411		Man5-9; Hy; A1G1S1; A2G2S1; A2G2S2			
					Asn3465		A2G2S1; A2G2S2			
					Asn3895		A2G2S2; A3G3S3			
					Asn4237		A2G2S1; A2G2S2			
					Asn4431		A2G2S1; A2G2S2			
Apolipoprotein D	P05090	Cholesterol transport (HDL)	0.1	0.1	Overall		A2FG2S2; A3G3S3	Conc↑	Conc↑(Females)	[59, 60, 62, 67, 91–93]
					Asn65		A2G2S2; A3G3S2; **A3G3S3**; A4G4S4			
					Asn98		A2G2FS1; A2G2S2; **A2FG2S2**; A3FG3S2; A3FG3S3; A4FG4S3			
Apolipoprotein F	Q13790	Cholesterol transport (VLDL)	0.073–0.096 (F-M)	0.0845	Overall					[94–96]
		Proprotein			Asn118		Man5-9			
		Proprotein			Asn139		Man5-9			
					Asn267					
Beta-2-glycoprotein 1	P02749	Scavenger of negatively charged compounds	0.2	0.2	Overall		A2G2S2 (58 %); A3G3S3 (28.5 %); A2G2S1 (6.5 %); A3G3S2 (5 %)	Conc↓, A3S↓, A2G2S2↑	Conc↑	[59, 97–102]
					Asn162		A2G2S2 (67 %); A3G3S3 (22 %); A2G2S1 (5 %); A3G3S2 (3 %)	A3↓, A2↑		
					Asn183		A2; A3			
					Asn193		A2G2S2 (49 %); A3G3S3 (35 %); A2G2S1 (8 %); A3G3S2 (7 %)	A3↓, A2↑		
					Asn253		A2; A3			
Ceruloplasmin	P00450	Copper dependent iron oxidation (Fe2+ to Fe3+)	0.15–0.96	0.355	Overall		A2G2S2 (62.75 %); FA2G2S2 (14.5 %); A3G3S3 (13.5 %); A3G3FS3 (7.25 %)	Conc↑		[59, 103–105]
					Asn138		**A2G2S2** (49 %); FA2G2S2 (26 %); A3G3S3 (12 %); A3FG3S3 (10 %); FA3FG3S3 (3 %)			
					Asn227	0 %	---			
					Asn358		**A2G2S2** (83 %); FA2G2S2 (12 %); A3G3S3 (5 %)			
					Asn397		**A2G2S2** (73 %); A3G3S3 (17 %); A3FG3S3 (6 %); FA2G2S2 (4 %)			
					Asn588		---			
					Asn762		**A2G2S2** (46 %); A3G3S3 (20 %); FA2G2S2 (16 %); A3FG3S3 (13 %); FA3FG3S3 (2 %); A4G4S4 (1 %); A4FG4S4 (1 %)			
					Asn926	0 %	---			
Fibrinogen		Coagulation (platelet aggregation)	2.0–4.5	3	Overall		A2G2S1(6) (53 %); A2G2S2(6) (33 %)			[59, 60, 67, 70, 106–117]
Fibrinogen alpha-chain	P02671				Asn453	0 %	---			
					Asn686	0 %	---			
Fibrinogen beta-chain	P02675				Asn394		**A2G2S1(6)**; A2G2S2(6)			
Fibrinogen gamma-chain	P02679				Asn78		**A2G2S1(6)**; A2G2S2(6)			
					Asn334		**A2G2S1(6)**; A2G2S2(6)	Glycosylation↑ in mutants		
Haptoglobin	P00738	Scavenger of hemoglobin	0.8–2.5	1.32	Overall		A2G2S2 (45 %); A2G2S1 (26 %); A3G3S3 (9 %); A3FG3S3 (6 %); A3G3S2 (5 %); A3G3S1 (5 %); A2FG2S1 (2 %); A2FG2S2 (1 %);	Conc↑, glycosylation complex, branching↑, F↑		[13, 118–126]
					Asn184	97.7 %	A2G2S2 (46 %); A2G2S1 (38 %); A3G3S3 (4 %); A3G3S2 (3 %); A3G3S1 (2 %); A2FG2S2 (3 %); A2FG2S1 (3 %); A3FG3S3 (1 %)			
					Asn207	97.4 %	A2G2S2 (47 %); A2G2S1 (39 %); A3G3S1 (7 %); A4G4S1 (2 %); A3FG3S1 (2 %); A4G4S2 (1 %); A2FG2S1 (1 %); A2FG2S2 (1 %)	S↑		
					Asn211	98.5 %	A2G2S2 (40 %); A3G3S3 (29 %), A3FG3S3 (21 %); A3G3S2 (10 %)	S↑		
					Asn241	95.8 %	A2G2S2 (47 %); A2G2S1 (26 %); A3G3S1 (10 %); A3G3S2 (8 %); A3G3S3 (4 %); A2FG2S1 (2 %); A2FG2S2 (1 %); A3FG3S2 (1 %); A4G4S1 (1 %);			
Hemopexin	P02790	Heme scavenger	0.4–1.5	0.8	Overall		A2G2S2 (90 %); FA2G2S2 (5 %)	Conc↑, Antennarity↑, F↑	Conc↑	[59, 60, 62, 67, 68, 70, 121, 127–134]
					Asn64		A2G2S2(6) (90 %); FA2G2S2 (5 %)			
					Asn187		A2G2S2(6) (90 %); FA2G2S2 (5 %)			
					Asn240					
					Asn246					
					Asn453		A2G2S2(6) (90 %); FA2G2S2 (5 %)			
Histdine-rich glycoprotein	P04196	Immunity, coagulation and angiogenesis regulator	0.1–0.15	0.125	Overall		A3-A4?	Conc↓	Conc↑	[60, 70, 135–138]
					Asn63					
					Asn125					
					Asn344					
					Asn345					
Kininogen-1	P01042	Coagulation	0.055–0.090	0.0725	Overall		A3-A4?, core F	Sialyl-Lewis X↑		[59, 60, 62, 67, 70, 139, 140]
					Asn48		core F			
					Asn169					
					Asn205		core F			
					Asn294		core F			
Serotransferrin	P02787	Iron transport	2–3	2.5	Overall		A2G2S2 (96.5 %); FA2G2S2 (2.5 %); A3G3S2 (1 %)	Conc↑↓ (pregnancy↑, inflammation↓), A3↑(hepatoma), glycosylation changed (CDGs, alcohol↓)	Conc↑ (stable from 2yo)	[8, 59, 141–151]
					Asn432		A2G2S2 (93.5 %); A3G3S2 (2.5 %); A2G2S1 (2.4 %); A2FG2S2 (1.6 %)			
		Minor Asn-X-Cys site			Asn491	2 %	A2G2S2 (100 %)			
					Asn630		A2G2S2 (85.9 %); FA2G2S2 (6.9 %); A2FG2S2 (2.8 %); A2G2S1 (2.2 %); A3G3S2 (1.0 %); FA3G3S2 (0.9 %); FA2FG2S2 (0.3 %)			
Vitronectin	P04004	Cell adhesion, coagulation	0.2–0.4	0.3	Overall		A2G2S2 (57 %); A3G3S3 (14.3 %); A3FG3S3 (10 %); A2G2S1 (8.7 %); Hy (6 %); FA2G2S2 (3.3 %)	Conc↑↓ (inflammation↑, liver fibrosis↓), hybrid↑(HCC), F↑ (HCC)		[17, 59, 67, 70, 152–154]
					Asn86		A2G2S2 (45 %); A3G3S3 (33 %); A3FG3S3 (20 %)			
					Asn169		A2G2S2 (76 %); Hy (18 %); A2G2S1 (6 %)			
					Asn242		A2G2S2 (50 %); A2G2S1 (20 %); FA2G2S2 (10 %); A3G3S3 (10 %); A3FG3S3 (10 %)			
Zinc-alpha-2-glycoprotein	P25311	Fat metabolism	0.05	0.05	Overall		A2G2S2 (97 %); A2G2S1 (3 %)	Conc↑ (cancer)		[59, 60, 67, 70, 96, 129, 155–158]
					Asn109					
					Asn112		A2G2S2 (100 %)			
					Asn128		A2G2S2 (100 %)			
					Asn259		A2G2S2 (90 %); A2G2S1 (10 %)			
Immunoglobulin A		Immunity (mucosal; FcaRI)	2.62	2.62	Overall		A2G2S2 (24 %); A2G2S1 (20 %); FA2BG2S2 (14 %)			[159–165]
IgA1	P01876			0.9	Asn144		A2G2S1 (50 %); A2G2S2 (27 %); A2BG2S1 (10 %)			
					Asn340		FA2G2S2 (46 %); FA2BG2S2 (40 %); FA2G2S1 (13 %)			
IgA2	P01877			0.1	Asn47					
					Asn92					
					Asn131					
					Asn205					
					Asn327					
Immunoglobulin D	P01880	Immunity	0.03; <0.003–0.4	0.035	Overall		Man8 (14.4 %); Man9 (13.5 %); FA2G2S2 (7.6 %); FA2G2S1 (7.3 %); FA2BG2S2 (6.5 %); A2G2S1 (6.1 %)			[166–170]
					Asn225		Man8; Man9			
					Asn316		FA2G2S2; FA2G2S1; FA2BG2S2; A2G2S1			
					Asn367		FA2G2S2; FA2G2S1; FA2BG2S2; A2G2S1			
Immunoglobulin E	P01854	Immunity (parasitic infection; allergy)	0.0003	0.0003	Overall		FA2G2S2 (25 %); FA2G2S1 (14.5 %); FA2BG2S2 (13.5 %); FA2BG2S1 (13.5 %); Man5 (8.5 %)	B↓, A3↓		[169, 171–176]
					Asn21		FA2G2S1 (30 %); FA2BG2S1 (30 %); FA2G2S2 (15 %); FA2BG2S2 (10 %)			
					Asn49		FA2G2S2 (30 %); FA2G2S1 (18 %); FA2BG2S2 (15 %); FA2BG2S1 (15 %)			
					Asn99		FA2G2S2 (40 %); FA2G2S1 (20 %)			
					Asn146		FA2G2S2 (50 %); FA2BG2S2 (30 %); FA2G2S1 (10 %)			
					Asn252		FA2BG2S1 (35 %); FA2BG2S2 (25 %); FA2G2S2 (15 %); FA2G2S1 (10 %)			
					Asn264	0	---			
					Asn275		Man5 (50 %); Man6 (15 %); Man7 (10 %); Man8 (10 %); Man9 (5 %)			
Immunoglobulin G		Immunity (primary; secondary; complement system)	7–18	11.8	Overall		FA2G1 (31 %); FA2G2 (23 %); FA2G2S1 (13 %); FA2 (10 %); FA2BG1 (5 %)	Conc↓(pregnancy), FA2↑, FA2G2↓(RA, CD…), G↑ + S↑(pregnancy), G↓	G↓	[19, 20, 24, 28, 33, 177–200]
Immunoglobulin G1	P01857		5.03	5.03	Asn180					
Immunoglobulin G2	P01859		3.42	3.42	Asn176					
Immunoglobulin G3	P01860		0.58	0.58	Asn227					
			0.58	0.58	Asn322					
Immunoglobulin G4	P01861		0.38	0.38	Asn177					
Immunoglobulin M	P01871	Immunity (complement system)	0.5–2.0	1.47	Overall		FA2BG2S1 (26 %); FA2G2S1 (19 %); Man6 (10 %); Man5 (6 %)			[159, 201–203]
					Asn46		FA2BG2S1; FA2G2S1			
					Asn209		FA2BG2S1; FA2G2S1			
					Asn272		FA2BG2S1; FA2G2S1			
					Asn279		Man5; Man6			
					Asn439	17	Man6; Man7; Man8			

Uniprot number, main role, concentrations, site occupancy and glycan composition of the abundant plasma glycoproteins as reported in the listed literature

The *N*-glycosylation of the proteins is reported both on a general level and, where available, with site specific information about glycan composition, glycan structure and site occupancy. Protein concentrations were taken from large studies when available and a mean value was calculated from the reported ranges otherwise. The general Oxford notation was used for naming the glycan structures, M for the unassigned high-mannose structures and Hy for hybrid structures. For details on the calculation see Supplementary Table 1

Throughout the text, Oxford nomenclature has been used to annotate individual glycan structures or compositions with A giving the number of antennae, F for the fucose (location specific), B for bisecting *N*-acetylglucosamine, G for galactoses and S for sialic acids. The number directly after the letter indicates the quantity of the specific features and the number in parenthesis its linkage. UniProt numbering was used for sequence and site identification.

## Alpha-1-acid glycoprotein (P02763; P19652)

Alpha-1-acid glycoprotein (AGP), also known as orosmucoid-1, is a 201 amino acid glycoprotein, which includes an 18 amino acid signal peptide. The molecular weight of the bare protein is 23.5 kDa, but the carbohydrate content leads to observed masses around 41–43 kDa [44]. Two isoforms are found in plasma (AGP1 and AGP2 encoded by *ORM1* and *ORM2* respectively), differing in 22 amino acids [44]. The protein is expressed by the liver and secreted in a monomeric form into the circulation, where it is observed in concentrations between 0.36 and 1.46 mg/mL with a mean of 0.77 mg/mL, men having slightly higher levels than women [204, 205]. The concentration of AGP has been reported to increase with age in females but not in males. Being an acute phase protein, its serum concentration rises in response to inflammatory stimuli, potentially increasing the concentration two- to four-fold [205].

The main functions of AGP are acute phase negative modulation of the complement system and transport of lipophilic compounds, both of these heavily modulated by the glycosylation of the protein [206, 207]. The immunomodulatory function is expected to be via interaction with selectins at a given site of injury (with sialyl-Lewis X as ligand), and inhibiting local complement deposition by charge and receptor competition [207]. As AGP may be used to transport lipophilic and acidic drugs to a site of injury, it is regarded as a good target for therapeutic development [206].

### Glycosylation

AGP has five *N*-linked glycosylation sites, namely Asn33, Asn56, Asn72, Asn93 and Asn103. Overall, the glycosylation was determined to mainly consist of fully sialylated tri- and tetraantennary structures, with potential antennary fucosylation in the form of sialyl-Lewis X [44, 45]. Site specific glycosylation was determined by high-performance (HP) liquid chromatography (LC)-electrospray ionization (ESI)-mass spectrometry (MS) and matrix assisted laser desorption/ionization (MALDI)-time-of-flight (TOF)-MS of tryptic glycopeptides [46]. Asn33 mainly contains the triantennary structure A3G3S3 (60 %) together with its antennary fucosylated variant A3FG3S3 (20 %), as well as some non-complete sialylated glycoforms (A3G3S2, 12.5 %). Asn56 contains similar structures (A3G3S3, 55 %; A3FG3S3, 12.5 %; A3G3S2, 10 %) and a fraction of diantennary glycans (A2G2S2, 22.5 %). Asn72 is occupied by a higher level of antennarity, having, next to its triantennary glycans (A3G3S3, 15 %), a number of tetraantennary compositions (A4G4S4, 30 %; A4G4S3, 15 %; A4G4S2, 10 %; A4FG4S3, 5 %; A4FG4S4, 5 %). A similar situation is seen at Asn93 (A4G4S4, 22.5 %; A4G4S3, 20 %; A3G3S3, 17.5 %; A4FG4S4, 7.5 %; A4FG4S3, 7.5 %; A3FG3S3, 7.5 %; A4G4S2, 7.5 %) and Asn103 (A4G4S4, 45 %; A3G3S3, 20 %; A4G4S2, 10 %; A4G4S3, 7.5 %; A3FG3S3, 5 %) [45, 46] (Table 1).

The glycosylation of AGP changes considerably with varying conditions. For instance, during the early stages of an acute-phase immune response the levels of fucosylated glycans (sialyl-Lewis X) increase significantly [45, 47–49], which continues to increase throughout the acute phase immune response [50]. In rheumatoid arthritis both fucosylation and sialylation have shown to increase significantly [51].

## Alpha-1-antitrypsin (P01009)

Alpha-1-antitrypsin (AAT), also known as alpha-1-protease inhibitor, alpha-1-antiproteinase or serpin A1, consists of 418 amino acids (including a 24 amino acid signal peptide) with an apparent mass of 51 kDa (including glycosylation). It is mainly produced in the liver by hepatocytes, but is also synthesized in monocytes, intestinal epithelial cells, and in the cornea [52, 208–211]. Due to its small size and polar properties, the glycoprotein can easily move into tissue fluids [52]. In healthy individuals, a plasma level of approximately 1.1 mg/mL is found, but the concentration can increase three- to four-fold during inflammation [212–215]. AAT occurs as three different amino acid sequences, of which the first is set as the standard sequence. Form 2 differs in the amino acid sequence 356–418 and form 3 lacks the amino acid sequence 307–418.

AAT inhibits a wide range of serine proteases, protecting tissues from enzymatic attacks [216]. Neutrophil elastase is its prime target, thereby preventing proteolytic destruction of elastase in the tissue of the lower respiratory tract (emphysema) [217]. It has been shown that AAT has anti-inflammatory properties and therefore it could potentially be used as a therapeutic agent for rheumatoid arthritis and type 1 diabetes [218, 219].

### Glycosylation

Three *N*-glycosylation sites have been identified on AAT, located at Asn70, Asn107 and Asn271 [52–54]. MALDI-TOF-MS analysis on released glycans revealed mainly di- and triantennary complex type species. Isoelectric focusing furthermore revealed eight different charge isoforms of AAT, of which isoform 4 (M4) and isoform 6 (M6) were the most abundant ones. Of M4, the most pronounced glycans were diantennary disialylated (A2G2S2) and triantennary trisialylated (A3G3S3) with a ratio of 2:1. Isoform M6 was mainly occupied with A2G2S2 structures [55].

LC-MS/MS analysis on tryptic glycopeptides treated with various specific exoglycosidases enabled a precise determination of the glycosylation in a site-specific manner [54]. Asn70 and Asn271 mainly contain diantennary disialylated (A2(2)G2(4)S2(6)) structures (91.3 and 99.3 % respectively), while core fucosylation (F(6)A2(2)G2(4)S2(6)) is less abundant (8.6 and 0.7 % respectively). Asn107 shows the highest variability of the sites, containing diantennary disialylated species A2(2)G2(4)S2(6), 52.5 %, with possible core fucosylation F(6)A2(2)G2(4)S2(6), 1.5 %), and 29.5 % triantennary trisialylated species A3(2,4,2)G3(4)S3(6,3,6)) with possible antennary fucosylation (A3(2,4,2)F(3)G3(4)S3(6,3,6), 16.7 % (Table 1). In addition, a small fraction of Asn107 is tetraantennary fully sialylated with potential antennary fucosylation. Interestingly, the diantennary structures contained mainly (α1-6-linked) core-fucosylation, while on triantennary structures the fucose was mainly detected as sialyl-Lewis X on the β1-4-linked *N*-acetylglucosamine of the α1-3-arm [54].

In a non-site-specific study, the glycosylation of AAT has been associated with physiological parameters such as BMI, cholesterol, glucose and insulin level. The same study showed that the changes in glycosylation could be found related to age and sex [56]. Furthermore, additional AAT isoelectric isoforms were identified in CDG-I (CDG-Ia and CDG-Ic) *i.e.* non-, mono- and diglycosylation across the three sites. A clear pattern could be found for which sites were occupied, as only Asn70 was occupied in the monoglycosylated form, and Asn70 and Asn271 were occupied in the diglycosylated isoform [57].

## Alpha-1B-glycoprotein (P04217)

Alpha-1B-glycoprotein (A1BG) is a 474 amino acid polypeptide with an apparent mass of 63 kDa (including glycosylation) [58]. The protein consists of five repetitive domains that show high homology with known immunoglobulin heavy and light chain variable domains, making the protein part of the immunoglobulin superfamily. A1BG is mainly produced in the liver, and is secreted to plasma to levels of approximately 0.22 mg/mL [58, 220]. The overall function of the protein is still unknown, but it has been found to bind cysteine-rich secretory protein 3 (CRISP3) [221], and has been associated with breast, liver, pancreas and bladder cancer, as well as with steroid-resistant nephrotic syndrome [222–226]. In addition, it has recently been proposed as an autoantigen in rheumatoid arthritis [227].

### Glycosylation

In A1BG, the *N*-glycosylation consensus motif (Asn-X-Ser/Thr) has been found at four locations Asn44, Asn179, Asn363, and Asn371 [58]. The occupancy of these sites has been verified by deglycosylated peptide LC-MS(/MS) and LC-Fourier transform ion cyclotron resonance (FTICR)-MS, but degrees of occupancy remain unknown [59, 60]. In addition, overall or site-specific glycosylation analysis also has not been performed for A1BG as of yet, although one source reports blood derived high-density lipoprotein (HDL)-associated A1BG Asn363 to be (at least) glycosylated with diantennary nonfucosylated monosialylated species [61]. Also, while not necessarily predictive for plasma glycoprotein glycosylation, in cerebrospinal fluid (CSF) Asn44 was shown to contain nonfucosylated di- (96 %) and triantennary (4 %) structures with at least one sialic acid [62]. Little is known about the changes in glycosylation of A1BG with disease.

## Alpha-2-HS-glycoprotein (P02765)

Alpha-2-HS-glycoprotein (A2HSG), also known as fetuin-A, alpha-2-Z-globulin, ba-alpha-2-glycoprotein and alpha-2-Heremans-Schmid-glycoprotein, is a 367 amino acids (18 amino acid signal peptide), 51–67 kDa glycoprotein [63, 64, 228]. It is built up from an A-chain (282 amino acids) and B-chain (27 amino acids) with a linker sequence (40 amino acids) [135, 229]. Originating from the liver, the protein is found at plasma levels of 0.3–0.6 mg/mL [229]. A2HSG acts at several sites and in a wide variety of (patho)physiological processes in the human system. Prominent functions include the scavenging of phosphate and free calcium, thereby preventing calcification, as well as binding and protecting matrix metalloproteases. In addition, the protein is known to bind the insulin receptor [230–233].

Increased levels of A2HSG are associated with obesity and type 2 diabetes mellitus [234]. On the other hand, decreased levels of A2HSG are found to cause several negative growth effects [230]. Furthermore, the protein has shown to protect a fetus from the maternal immune system by inhibition of tumor necrosis factor [231, 235]

### Glycosylation

The A-chain of A2HSG contains two *N*-glycosylation sites at Asn156 and Asn176, as well as two *O*-glycosylation sites at Thr256 and Thr270 [63]. The B-chain contains one core 1 *O*-glycan on Ser346, and no *N*-glycans [64]. Exoglycosidase treatment has reported 6.2 sialic acids to be present per A2HSG molecule, of which 2.5 are α2-3-linked and 3.7 are α2-6-linked [65]. Sequentially, four galactoses in β1-4-linkage were released from *N*-acetylglucosamines, pointing towards two diantennary *N*-glycans in addition to the *O*-glycosylation [65]. LC-ESI-MS experiments have confirmed these findings, reporting around 96 % A2G2S2 glycosylation to be present on A2HSG [66]. Furthermore, low levels of fucosylated glycans were observed, at least on Asn156 [66, 67].

Differential abundance of Asn156 glycopeptides has been shown in pancreatic cancer and pancreatitis, with increased levels of fully sialylated triantennary glycans with or without fucose, and a decrease in the A2G2S2 structure in pancreatitis [68].

## Alpha-2-macroglobulin (P01023)

Alpha-2-macroglobulin (alpha2M), also known as C3 or PZP-like alpha-2-macroglobulin domain-containing protein 5, is a 1474 amino acid (23 amino acid signal peptide) 720 kDa (glycosylated) glycoprotein consisting of four similar 180 kDa subunits (160 kDa without glycosylation) which are linked by disulfide bridges [69]. It is produced by the liver and present at plasma levels of approximately 1.2 mg/mL [236]. The main function of alpha2M is to bait and trap proteinases [69]. To do this, the protein contains a bait peptide sequence known to interact with many common plasma proteases such as trypsin, chymotrypsin, and various others in the complement system. Upon proteolysis, a conformation change in alpha2M traps the causative protease and the complex is subsequently cleared from the plasma [237–239].

### Glycosylation

Eight *N*-glycosylation sites have been identified on each alpha2M subunit at Asn55, Asn70, Asn247, Asn396, Asn410, Asn869, Asn991 and Asn1424 [59, 60, 67, 69–71]. The total pool of alpha2M-derived glycans was analyzed by LC-fluorescence with exoglycosidase digestion. This revealed a high abundance of diantennary structures, both non-fucosylated (55 %) and core-fucosylated (30 %), which are mainly mono- and disialylated (A2G2S2, A2G2S1, FA2G2S2, FA2G2S2) [72]. In addition, Man5-7 type structures was detected as well (8 %) as species with a lower degree of galactosylation and sialylation. Low levels of triantennary structures have also been identified [72].

Interestingly, the high-mannose type glycans have been shown to specifically occur at Asn869 with a relative abundance of approximately 70 %, the other 30 % being FA1G1S1 [72]. This high-mannose type glycosylation is likely the means by which alpha2M interacts with mannose-binding lectin (MBL) to target proteases present on the surface of invading microorganisms [72]. The Asn869 occupancy ratio suggests that each alpha2M tetramer contains three oligomannose glycosylated Asn869 sites and one FA1G1S1, although this is speculative [72]. The other *N*-glycosylation sites mainly contain complex type glycans and glycoproteomic analysis suggests that the core-fucosylated species are present to at least some degree at specific sites Asn55 and Asn1424 [67].

Changes in the glycosylation of alpha2M have been associated with autoimmune diseases and cancer. Site occupancy in particular has been linked with systemic lupus erythematosus, while a compositional change has been described in multiple sclerosis [73, 74].

## Antithrombin-III (P01008)

Antithrombin-III (AT-III, generally referred to as antithrombin), encoded by the *SERPINC1* gene, is a single chain 464 amino acid (32 amino acid signal peptide) protein of approximately 58 kDa, of which 17 % are carbohydrates [240–242]. It is part of the serine protease inhibitor family. The concentration of antithrombin in blood was found to be 0.15 mg/mL [243]. The protein can be found in an α and β form which differ in the number of occupied glycosylation sites and of which α is 10–20 times more abundant [75]. Antithrombin participates in the regulation of blood coagulation by inactivating thrombin, factor IXa, Xa, XIa, XIIa, and other serine proteases [244]. Its function is enhanced by heparin and heparan sulfate [76, 245, 246]. Several thrombosis disorders are associated with antithrombin deficiency (ATD), both inherited and acquired. Type I ATD shows reduced concentrations of antithrombin, while type II ATD generally shows normal concentrations with reduced heparin binding and thus lower functionality [247].

### Glycosylation

The sequence of antithrombin shows four potential *N*-glycosylation sites: Asn128, Asn167, Asn187 and Asn224. The α form is fully glycosylated, while the β form is not glycosylated at Asn167 [77, 78]. The β form binds heparin more efficiently and thus shows an enhanced anticoagulant effect. Several studies suggest that the Asn-X-Thr motif of Asn128, Asn187 and Asn224 are in general glycosylated more easily than the Asn-X-Ser motif of Asn167 [79–81].

The glycans present on AT-III are mainly of the diantennary complex type without core fucose, bearing one (0–30 %) or two (70–100 %) α2-6-linked sialic acids, as it was established using chemical and enzymatic methods [82, 83]. Using MALDI and LC-ESI-MS these findings have been confirmed in a site-specific manner [75, 76, 84, 85]. β-AT was exclusively decorated with three diantennary fully sialylated structures (A2G2S2, 4.2 %), with trace amounts of core fucose on one of the glycan (FA2G2S2, 1.3 %). At Asn128 and Asn224 of α-AT, only the A2G2S1 and A2G2S2 structures were identified. At Asn167, occupied only in the α form of AT-III, A3G3S3 has additionally been detected, whereas Asn187 showed the most variability, bearing also a minor amount of fucose (FA2G2S2). Furthermore, at Asn187 some A3G3S2 has been observed. All glycoforms other than A2G2S2 are mentioned to be minor, although no relative or absolute quantification has been performed [75, 76, 84, 85].

A mutation associated with type II antithrombin deficiency (K241E), although not adjacent to a glycosylation consensus sequence, was found to result in decreased heparin binding due to the presence of core fucose [86].

## Apolipoprotein B-100 (P04114)

Apolipoprotein (Apo) B-100 is a 550 kDa 4560 amino acid protein (4536 amino acids without the signal peptide, corresponding to a theoretical mass of 513 kDa without glycosylation) found in low density and very low density lipoproteins (LDL and VLDL) [248, 249]. A shorter isoform found in chylomicrons, named Apo B-48, is coded by the same gene, but contains only 48 % of Apo B-100 sequence [250, 251]. Apo B-100 is exclusively synthetized by the liver, while Apo B-48 is synthetized in the small intestine [252]. Apo B-100 is found in plasma at concentrations of approximately 0.5 mg/mL (0.88 to 0.97 mmol) [34, 212, 253, 254]. The protein has a major role in the assembly of VLDL and lipoproteins, and transports the majority of plasma cholesterol [255–257]. It can be covalently linked to Apo A to form the lipoprotein(a) particle. Apo A itself is a low abundant plasma glycoprotein possessing one *N*-glycosylation site located at Asn263 (mainly occupied by diantennary mono- and disialylated *N*-glycans) [87, 258].

In coronary heart disease, the ratio of LDL-Apo A/B-100 is used for estimating the risk of acute myocardial infarction [253]. Apo B-100 and Apo B-48 mutations caused by *APOB100* and *MTP* (microsomal triglyceride transfer protein) gene defects are associated with metabolic disorders like abetalipoproteinaemia, hypobetalipoproteinemia and hypercholesterolemia [259–261].

### Glycosylation

Apo B-100 is highly glycosylated, and contains 19 potential *N*-glycosylation sites located at Asn34, Asn185, Asn983, Asn1368, Asn1377, Asn1523, Asn2239, Asn2560, Asn2779, Asn2982, Asn3101, Asn3224, Asn3336, Asn3358, Asn3411, Asn3465, Asn3895, Asn4237 and Asn4431. Of these, 17 are reported occupied by diantennary complex type glycans, as well as by high mannose and hybrid type structures [11, 87]. LC-fluorescence with exoglycosidase digestion has revealed the major glycans to be A2G2S1(6) (29.2 %), A2G2S2(6) (23.6 %), A2G2 (7.2 %), Man9 (8.6 %) and Man5 (6.9 %) [87]. In addition, low levels of the Man6-8 have been reported. Most of the sialic acids were α2-6-linked (91 %) [87].

A site specific analysis of the glycosylation has been performed by LC-ESI-MS(/MS) on tryptic and chymotryptic glycopeptides [11]. It was shown that the high mannose type glycans were mainly present on sites Asn185, Asn1368, Asn1377, Asn3336 and Asn3358, while the complex type (mono- and disialylated diantennary) glycans were located at Asn983, Asn2239, Asn2779, Asn2982, Asn3101, Asn3224, Asn3465, Asn3895, Asn4237 and Asn4431. Ans3895 is exceptional in this regard, as triantennary compositions have been observed as well. The largest variation is present on sites Asn1523 and Asn3411, as these display oligomannose, hybrid and complex structures. Asn3411, the nearest *N*-glycosylation site to the receptor of the LDL-binding site shows degrees of fucosylation. Asn34 and Asn2560 are not reported to be glycosylated [11].

The role of the glycans structures in LDL and/or Apo B-100 has been examined in several studies but their exact function is still unknown, although its degree of sialylation might serve the atherogenic properties of LDLs [87–90].

## Apolipoprotein D (P05090)

Apolipoprotein D (Apo D), also referred to as thin line polypeptide, is a small glycoprotein of 189 amino acids (with a signal peptide of 20 amino acids), with a molecular weight varying between 19 and 32 kDa depending on its glycosylation [262, 263]. While it shares their name, it does in fact not resemble other apolipoproteins, and shares more homology with the lipocalin protein family [264]. It was originally assimilated to the apolipoprotein family due to its early association with lipid transport. Apo D is mainly synthetized in fibroblasts and to a lesser extent in the liver and intestine, where the other apolipoproteins are usually produced [265]. Its plasma levels are approximately 0.1 mg/mL [266, 267]. The common form of Apo D in plasma is a monomer, although it can also exist as a heterodimer linked to apolipoprotein A-2 via a disulfide bridge.

Apo D can form complexes with lecithin cholesterol acyltransferase and is implicated in the transport and transformation of lipids [264, 268–270]. It has been reported to have a potential role in colorectal cancer [265]. In addition, the protein is present at high concentrations in the cyst fluid where its concentration can be 500 times higher than in plasma, which can be associated with an increased risk of breast cancer [271–273]. Apo D has a tendency to accumulate in CSF and peripheral nerves of patients with Alzheimer’s disease and other neurodegenerative conditions [274, 275]. A positive correlation between age and Apo D levels has been reported in females, but not in men [276, 277].

### Glycosylation

Two glycosylation sites have been reported and confirmed for Apo D, namely Asn65 and Asn98 [59, 60, 91]. These are mainly occupied by complex type *N*-glycans ranging from diantennary to tetraantennary structures, with potential elongation of the antennae in the form of *N*-acetyllactosamine (LacNAc) repeats [91]. LC-MS with exoglycosidase digestion has revealed the most abundant glycoforms per site as well. Asn65 mainly contains nonfucosylated triantennary structures with full sialylation (A3G3S3), less abundant signals including di- and tetraantennary species with high degrees of sialylation (A2G2S2; A4G4S4). Contrarily, Asn98 predominantly contains fucosylated species, also ranging from di- to tetraantennary, here the main signal being diantennary (A2FG2S2) [62, 67, 91, 92]. Treatment with β-galactosidase failed to trim one antenna of its galactosylation, strongly suggesting the presence of the antennary fucosylation, known to prevent this digestion [91, 93]. Studies have shown the implication of Apo D in conditions like Alzheimer’s disease but no information about the role of glycosylation has been reported yet.

## Apolipoprotein F (Q13790)

Apolipoprotein F (Apo F), also called lipid transfer inhibitor protein (LTIP), is a glycoprotein with an apparent mass of 29 kDa. The polypeptide chain of 326 amino acids is processed, with the first 165 amino acids being the signal peptide and the propeptide, resulting in a theoretical mass of 17.4 kDa for the peptide backbone of the mature protein [278]. Apo F is expressed in the liver and is secreted in plasma to concentrations of 0.07 mg/mL in females and 0.10 mg/mL in males [94, 278, 279]. The protein regulates cholesterol transport, inhibits cholesteryl ester transfer protein (CETP), and is found in combination with lipoproteins of all subclasses (high density, low density and very low density lipoproteins (HDL, LDL, VLDL)) as well as with apolipoproteins A1 and A2 [279, 280].

### Glycosylation

Three potential *N*-glycosylation sites of Apo F are located at Asn118, Asn139 and Asn267, as well as one *O*-glycosylation site at Thr291 [94, 95]. Asn118 and Asn139 are glycosylated with high-mannose structures, as proven by exoglycosidase treatment, but will not contribute to plasma glycosylation as they are part of the proprotein [95]. Asn267, on the other hand, is not sensitive to this treatment, suggesting that it would contain complex-type *N-*glycans [95]. The presence of sialic acids on the protein has been indicated by sialidase treatment with western blotting as readout [94]. However, as *O*-glycanase has also shown the presence of *O*-glycans on the protein, it is unclear whether the sialylation arises from the *N*- or *O*-glycosylation [94]. In CSF, the *O*-glycans on Thr291 are reported to be of core 1 or 8 type [96]. No disease-related information is available with regard to the glycosylation of Apo F.

## Beta-2-glycoprotein 1 (P02749)

Beta-2-glycoprotein 1 (B2GPI) is also called apolipoprotein H, APC inhibitor, activated protein C-binding protein, and anticardiolipin cofactor. It is a 50 kDa (including around 19 % carbohydrate content) 345 amino acid single polypeptide chain (with a signal peptide of 19 amino acid) belonging to the complement control protein (CCP) superfamily [97]. It consists of five similar CCP domains of approximately 60 amino acids [281]. B2GPI is mostly synthetized in hepatocytes and is found in blood at around 0.2 mg/mL [282]. The main function of B2GPI is the scavenging of negatively charged compounds such as DNA, sialylated glycoproteins, and (phospho)lipids, which may otherwise induce unwanted coagulation and platelet aggregation [283–285]. The precise binding properties of the protein depend on the conformation, *i.e.* open or closed, which is proposed to be dependent on the glycosylation [98, 99, 286].

The serum level of B2GPI increases with age, and is reduced during pregnancy and for patients suffering from stroke and myocardial infarctions [98, 287]. Additionally, it is the major antigen in antiphospholipid syndrome [98, 99].

### Glycosylation

B2GPI possesses four theoretical *N*-glycosylation sites at Asn162, Asn183, Asn193 and Asn253, as well as an *O*-glycosylation site at Thr149 [97, 98, 100, 101]. The *N*-glycosylation sites have been confirmed by crystallography (finding attached *N*-acetylglucosamines and mannoses) as well as by deglycosylated Lys-C peptide reverse phase (RP)-LC-MS after lectin capture [59, 101]. Generally, the glycosylation of B2GPI is of the di- and triantennary type containing high levels of sialylation, with minor amounts of fucosylation [99]. Site-specific information is available only for Asn162 and Asn193 [99].

Glycopeptide LC-ESI-quadrupole (Q)-TOF-MS revealed the glycosylation of Asn162 to be 67 % diantennary disialylated (A2G2S2) and 22 % triantennary trisialylated (A3G3S3), minor species including the di- and triantennary species lacking one sialic acid (5 and 3 % respectively) [99]. The Asn193 site showed the same compositions, but with a higher level of triantennary species (35 %) and a corresponding lower percentage of diantennary species (49 %). Minor species again include the incompletely sialylated variants (8 and 7 % for the di- and triantennary species respectively) [99]. The findings were confirmed by MALDI-QTOF-MS, although a lower degree of sialylation was observed. This difference is likely due to the tendency of MALDI ionization to induce in-source and metastable decay of sialylated glycan species [99, 102]. For the two noncharacterized *N*-glycosylation sites (Asn183 and Asn253) di- and triantennary glycans are expected as well, given the 19 % of the total protein weight being attributed to the carbohydrate content [101].

Patients suffering from antiphospholipid syndrome (APS) showed a decrease in the amount triantennary sialylated glycans, and thus a relative increase in diantennary fully sialylated ones. This effect was particularly pronounced for Asn162 [99].

## Ceruloplasmin (P00450)

Ceruloplasmin (CP), also called ferroxidase, is a 132 kDa (120 kDa without glycosylation) 1065 amino acid (19 of which are signal peptide) glycoprotein synthesized by the liver [103]. It consists of a single polypeptide chain, and belongs to the multicopper oxidase family [103]. Concentrations for CP range from 0.15 to 0.96 mg/mL with a mean of 0.36 mg/mL, while elevated levels have been reported upon inflammatory stimulation [34, 288, 289]. CP can bind six to seven atoms of copper, in this manner containing and transporting 95 % of the copper found in plasma. The main function of the protein, however, is in iron metabolism. CP has ferroxidase activity oxidizing Fe^2+^ to Fe^3+^ without releasing radical oxygen species, while also facilitating iron transport across the cell membrane [103].

### Glycosylation

Of the seven potential CP *N*-glycosylation sites Asn138, Asn227, Asn358, Asn397, Asn588, Asn762 and Asn926, four (Asn138, Asn358, Asn397, and Asn762) are confirmed to be glycosylated [59]. The remaining sites (Asn227, Asn588, and Asn926) are all in a β-strand within a hydrophobic region, potentially preventing site occupation [103]. NMR spectroscopy has revealed the overall CP glycan species to be sialylated diantennary A2(2)G2(4)S2(6) and sialylated triantennary A2(2,2,4)G4(4)S3(6,6,3/6). Partial core fucosylation has been found for the diantennary species, while of the triantennary species the α2-3-linked sialic acid-containing arm can be α1-3-fucosylated to form sialyl-Lewis X [104].

For the confirmed *N*-glycosylation sites, tryptic glycopeptide LC-ESI-MS(/MS) was used to study the site-specific glycosylation on a compositional level, and relatively similar ratios of di- and triantennary glycan species were found across the sites [105]. Asn138 is mainly occupied by the diantennary structures A2G2S2 (49 %) and FA2G2S2 (26 %), followed by the triantennary structures A3G3S3 (12 %) and A3FG3S3 (10 %). Small amounts of difucosylated species have been detected as well (FA3FG3S3, 3 %). Asn358 contains a higher abundance of diantennary species (A2G2S2 83 %, and FA2G2S2 12 %), the triantennary species A3G3S3 only accounting for 5 %. For Asn397 the main glycan is A2G2S2 (73 %), followed by A3G3S3 (17 %), A3FG3S3 (6 %) and FA2G2S2 (4 %). Analysis of Asn762 showed the main glycan to be A2G2S2 as well (46 %), with the additional compositions A3G3S3 (20 %), FA2G2S2 (16 %), A3FG3S3 (13 %), FA3FG3S3 (2 %), A4G4S4 (1 %) and A4FG4S4 (1 %) [105]. No information about the glycosylation of human ceruloplasmin in disease was found with the preparation of this review.

## Fibrinogen (P02671; P02675; P02679)

Fibrinogen is a 340 kDa glycoprotein that is synthesized in the liver by hepatocytes, and plays a key role in blood clotting [290, 291]. The protein consists of two sets of three different polypeptide chains named the α-chain (610 amino acids), β-chain (461 amino acids), and γ-chain (411 amino acids), arranged in a α_2_β_2_γ_2_ hexamer linked by disulfide bonds [106, 292, 293]. In plasma, fibrinogen is typically found at concentrations of 2–6 mg/mL with a mean of 3 mg/mL, with women having slightly higher levels, and it is also present in platelets, lymph nodes, and interstitial fluid [106, 293–296].

Fibrinogen is cleaved by thrombin into fibrin, one of the essential components of blood clots after injury [106, 291, 297]. Furthermore, it acts as a cofactor in platelet aggregation, assists rebuilding of epithelium, and can protect against infections in interferon γ (IFNγ)-mediated hemorrhage [106, 298, 299]. In addition, the protein can facilitate the immune response via the innate and T-cell pathways [300–303].

### Glycosylation

The α-chain of fibrinogen is not *N*-glycosylated, even though it harbors two potential *N*-glycosylation sites at Asn453 and Asn686. The β- and γ-chain are *N*-glycosylated at Asn394 and Asn78, respectively [106–108]. By MALDI-TOF-MS and HPLC with exoglycosidase digestion, the predominant glycan structures present on these chains were found to be A2G2S1 (53 %) and A2G2S2 (33 %). Sialic acids are mainly α2-6-linked, but a degree of α2-3-linkage has been reported as well depending on the source or analytical method [109, 110]. Bisecting *N*-acetylglucosamine and core fucosylation are found in minor quantities [110]. Comparisons between plasma and serum *N*-glycan profiles revealed that fibrinogen could contribute for 22 % to the total intensity of the diantennary monosialylated structures (A2G2S1) [110].

Site-specific analysis showed diantennary glycans with zero, one or two sialic acids on Asn394 (β-chain) and Asn78 (γ-chain) [107]. The glycosylation sites have been confirmed in studies at the level of deglycosylated glycopeptides, showing occupancy of Asn394 of the β-chain and Asn78 of the γ-chain, and surprisingly on the α-chain Asn686 as well [59, 60, 70, 108]. The β-chain glycosylation site has furthermore been observed in a core-fucose targeted study [67]. In addition to *N*-glycosylation, all fibrinogen chains may carry *O*-glycans [107].

The general degree of sialylation may be influencing the solubility of fibrinogen, and thereby play a crucial role in blood clotting processes resulting in different fiber structures. [111–115]. In the Asahi mutant of the γ-chain, Asn334 has been reported to contain an additional *N*-glycosylation site [116]. Patients exhibiting the Asahi variant of fibrinogen displayed abnormally long blood clothing time, suggesting that the effect induced by that extra glycosylation site disturbs the fibrin polymerization process [116, 117].

## Haptoglobin (P00738)

Haptoglobin (Hp) is a 406 amino acid (18 amino acid signal peptide) acute-phase glycoprotein with a peptide backbone of 45 kDa. It is synthesized in the liver by hepatocytes as a single polypeptide chain and is also found in skin [304, 305]. During its synthesis, Hp is cleaved into a light α chain and a heavy β chain that are connected via disulfide bonds. Two variants of the α chain originating from the sequence Val19-Gln160 and differing by the subsequence Glu38-Pro96 can exist, α^1^ having this subsequence once while α^2^ has it twice, resulting in α chains of 83 or 142 amino acids with a respective molecular mass of 9 and 16 kDa. The 40 kDa β chain is made of 245 amino acids originating from the sequence Ile162-Asn406 [306, 307]. The combination of different allelic variants of the α chain (α^1^ and α^2^) with β chain(s) creates the polymorphism observed in Hp. There are three major Hp phenotypes called Hp1-1, Hp2-1 and Hp2-2. They have a configuration of (α^1^β)_2_, (α^1^β)_2_ + (α^2^β)_n = 0, 1, 2, …_ and (α^2^β)_n = 3, 4, 5, …_, respectively, which are observed at different ratios among ethnicities [118, 308–310]. Caucasians have around 13 % of phenotype Hp1-1, 46 % of Hp2-1 and 41 % of Hp2-2. Hp is typically found at a plasma levels in the range of 0.6–2.3 mg/mL with a mean of 1.32 mg/mL [118]. Elevated Hp levels have been reported with inflammation and malignant diseases [308, 311, 312]. It should be taken into account that the concentration as well as the molecular mass including glycosylation may vary among phenotypes (86–900 kDa) [118]. The half-life of Hp is found to be on average four days.

The major function of Hp is to protect tissues from oxidative damage by capturing hemoglobin [307, 313]. It has been reported that Hp polymorphism has an effect on its physiological properties, for instance Hp1-1 binds hemoglobin stronger than Hp2-2 [314]. Certain diseases seem to be dependent on the polymorphism, as individuals with the Hp1-1 phenotype seem to have a higher concentration of induced antibodies in their plasma after vaccination, infections or liver diseases compared to the other phenotypes [118, 310].

### Glycosylation

Four *N*-glycosylation sites have been identified on the β-chain of Hp, located at Asn184, Asn207, Asn211 and Asn241 [119–121]. Analysis with (nano-)RPLC-ESI-MS/MS and MALDI-MS/MS of Hp glycopeptides (trypsin and GluC) revealed that all sites are occupied by complex type *N*-glycans [119, 120]. The site occupancy for Asn184 was determined at 97.7 %, Asn207 at 97.4 %, Asn211 at 98.5 % and Asn241 had a site occupancy of 95.8 %. Treatment with α2-3-sialidase showed that the sialic acids were mainly α2-6-linked, while β1-4-galactosidase treatment revealed that only antennary fucosylation was present, which was in agreement with the obtained collision-induced dissociation (CID) fragmentation spectra [120].

Two recent studies showed some discrepancies in the relative abundances for the identified sites. For example, Asn184 was found to contain mainly diantennary species with two sialic acids (A2G2S2, 88 and 46 %), followed by diantennary monosialylated (A2G2S1, 7 and 38 %) and triantennary disialylated (A3G3S2, 4 and 3 %) glycans. A low percentage of fucosylation was identified (A3FG2S2, 1 and 3 %; A3FG3S2, 0.3 and 1 %) [119, 120].

A possible reason for discrepancies at Asn207/Asn211 is that the first study did not differentiate the two *N*-glycosylation sites (Asn207 and Asn211) on the same peptide backbone that showed 7 different combinations. The major combinations were 1) one diantennary fully sialylated (A2G2S2) and one triantennary disialylated (A3G3S2, 45 %), 2) two diantennary disialylated glycans (A2G2S2, 30 %), and 3) one diantennary fully sialylated (A2G2S2) and one triantennary disialylated and fucosylated (A3FG3S2, 12 %). The combination of a diantennary monosialylated (A2G2S1) with a diantennary disialylated glycan (A2G2S2) accounted for 6 %, the diantennary and triantennary fully sialylated species (A2G2S2 and A3G3S3) for 5 %, and the remaining combinations accounted for approximately 1 % in total [119]. The second study reported the glycoforms for each site separately due to an additional GluC protease treatment. Asn207 seems to contain mainly A2G2S2 (47 %) and A2G2S1 (39 %), followed by A3G3S1 (7 %) next to some minor tetraantennary and fucosylated species. Interestingly, glycosylation site Asn211 appears to have a higher degree of triantennary species, with A2G2S2 (40 %), A3G3S3 (29 %), A3FG3S3 (21 %), and A3G3S2 (10 %) [120].

The two studies report that Asn241 carries mainly diantennary glycans, A2G2S2 being the most abundant variant with 87 and 47 % (values reported in the two separate studies), followed by A2G2S1 (4 and 26 %), A3G3S1 (n.d. and 10 %), A3G3S2 (6 and 8 %), A3G3S3 (n.d. and 4 %) and A2FG2S1 (<1 and 2 %). Low levels of tetraantennary species have been detected as well, with and without fucosylation varying from mono- to tetrasialylated [119, 120].

Both studies evaluated the glycosylation of Hp in patients with liver cirrhosis (LCH) and hepatocellular carcinoma (HCC). No difference in site occupancy could be observed between healthy and disease, but the number of detected glycoforms was increased (healthy 34 glycoforms, LCH 56 glycoforms, HCC 62 glycoforms) [120]. Increased branching and fucosylation were reported, with species carrying up to five fucoses [119, 121]. Furthermore an increase in sialylation was noticeable for the glycopeptide containing *N*-glycosylation sites Asn207 and Asn211 [119]. Those carbohydrate structures have been reported in another study along with some new ones but they were not quantified [13].

Furthermore, core-fucosylation was identified on the *N*-glycosylation site Asn184 [122]. Diantennary disialylated structures contained core-fucosylation (FA2G2S2) instead of antennary fucosylation. Several reports reveal that fucosylation plays an important role in many diseases such as pancreatic cancer, LCH and HCC [123, 124]. Another recent study examined the galectin-1 binding ability of Hp in the sera of metastatic breast cancer patients, where the binding was twice as strong, possibly due to a difference in glycoforms [125, 126].

It is interesting to see that two studies from the same year report different glycosylation patterns for Hp [119, 120]. This might be caused by a different ethnicity of the sample donors, as one study has been performed in China and the other in the United States, two geographical regions that have been reported to have different phenotype distributions [118]. That difference has not yet been taken into account in glycomics studies.

## Hemopexin (P02790)

Hemopexin (HPX), also known as beta-1B-glycoprotein, is a 462 amino acid (23 are part of the signal peptide) single polypeptide chain plasma glycoprotein with a peptide backbone of 51 kDa and an apparent mass ranging from 57 to 80 kDa depending on its glycosylation [315–317]. The protein is mainly expressed by the liver and found in serum at levels of 0.8 mg/mL in adults, while levels in newborns have been measured around 20 % of that value [318, 319]. It is also expressed in the central nervous system, in the retina and in the peripheral nerves. The protein structure is controlled by six disulfide bridges next to its glycosylation [316]. HPX is an acute phase response glycoprotein, capable of binding heme with the highest known affinity of all plasma proteins. When a heme is captured, the complex can be recovered from plasma by the HPX receptor (such as found on the membrane of liver parenchymal cells) leading to internalization, catabolization of the heme and recycling of the proteins involved. After the process, HPX is free to return to the circulation. HPX is found to be expressed in large quantities in case of inflammation, a state in which heme is highly abundant in plasma. As heme would otherwise induce oxidative stress, the function of HPX can be described as antioxidant [316].

### Glycosylation

HPX contains five confirmed *N*-glycosylation sites located at Asn64, Asn187, Asn240, Asn246 and Asn453 [59, 60, 70, 127–130]. In general, plasma HPX *N*-glycosylation consists mainly of diantennary structures with high levels of galactosylation, while low levels of triantennary and fucosylated structures have also been reported [67, 121, 131–133]. The degree of sialylation of the protein remains to be fully investigated, but lectin capturing of α2-6-sialylated HPX glycopeptides followed by LC-MS(/MS) analysis has revealed the presence of fully sialylated antennae and only low levels of monosialylated diantennary glycans [68]. Combining the information, the main glycan composition on HPX is expected to be A2G2S2. Site-specific characterization has been achieved on a compositional level for *N*-glycosylation sites Asn64, Asn187 and Asn453, each of them showing similar ratios of glycoforms (85–94 % diantennary nonfucosylated, 4–7 % diantennary fucosylated, as well as low levels of triantennary structures) [62, 121]. Asn240 and Asn246 remain uncharacterized, likely due to their close proximity. The antennarity and the degree of fucosylation (core and antennary) have been reported to increase with LCH and HCC [134]. Notably, HPX also contains two *O*-glycosylation sites (Thr24 and Thr29) one of which is located on the *N*-terminal threonine (after removal of the signal peptide), and a potential minor *O*-glycosylation in the Ser30-Thr40 region [62, 127, 130].

## Histidine-rich glycoprotein (P04196)

Histidine-rich glycoprotein (HRG), also called histidine-proline-rich glycoprotein (HPRG), has an apparent molecular mass of 72 kDa (peptide backbone of 60 kDa) and consists of 525 amino acid (507 without the signal peptide) [320, 321]. The protein occurs in plasma at concentrations of 0.1–0.15 mg/mL, and is mainly produced by the liver parenchymal cells although some reports suggest synthesis in immune cells as well [322–325]. Levels in newborns are only approximately 20 % of those in adults [326]. HRG is known to regulate immunity, coagulation and angiogenesis [327]. To achieve this, it interacts with many different ligands including heme, heparin, plasminogen, fibrinogen, thrombospondin and immunoglobulin G, as well as many cell surface receptors and divalent cations such as Zn^2+^ [325]. It is a negative acute phase protein, showing decreased plasma levels during inflammation, injury or pregnancy [328].

### Glycosylation

HRG is expected to have a large degree of glycosylation, as 14 % of the protein weight (around 10 kDa) has been attributed to the oligosaccharide portion [135]. Three *N*-glycosylation sites have been confirmed by glycoproteomic analysis, located at Asn63, Asn125 and Asn344 [60, 70]. Another glycosylation site is theoretically present at Asn345, but the direct vicinity with the site Asn344 may sterically hinder its occupation and additionally complicates its analysis. Interestingly, a common polymorphism can induce a new glycosylation site at Asn202 by replacing a proline by a serine at position 204, creating the motif Asn-X-Ser, and *N*-glycanase treatment revealed a mass difference of 2 kDa attributed to the new Asn202 carbohydrate compared to the unmodified form of HRG [136]. The sequence Asn87-Asp-Cys found in HRG has been reported to contain glycosylation (in bovine protein C) but no clear evidence of its presence has yet been made for human HRG [137, 138]. With regard to the classical sites, little is known, and to the best of our knowledge, neither site occupancy nor relative abundance of glycan structures have been studied. However, if a carbohydrate mass of 10 kDa needs to be distributed across three glycosylation sites, the average site would contain glycans of over 3300 Da (putting them into the tri- and tetraantennary range with high levels of galactosylation, sialylation and/or fucosylation). No reports about changes in glycosylation under disease conditions were found for HRG.

## Kininogen-1 (P01042)

Kininogen-1, also called alpha-2-thiol proteinase inhibitor, Fitzgerald factor, high-molecular-weight kininogen (HMWK) or Williams-Fitzgerald-Flaujeac factor, has a single polypeptide chain of 644 amino acid (18 belonging to the signal peptide) and approximates 114 kDa apparent molecular mass (while its theoretical mass without glycosylation is 70 kDa) [139]. Kininogen can be cleaved into six different subchains called kininogen-1 heavy chain, T-kinin (Ile-Ser-bradykinin), bradykinin (kallidin I), lysyl-bradykinin (kallidin II), kininogen-1 light chain, and low molecular weight growth-promoting factor. In its intact form, the protein is a cysteine proteinase inhibitor, implicated in blood coagulation and inflammatory response and it can bind calcium, while the individual subchains can have many other functions [329–332]. Kininogen is mainly synthetized in the liver to plasma concentrations of 55–100 μg/mL, where it is mostly found in complex with prekallikrein or factor XI to position the coagulation factors near factor XII [331, 333–336].

### Glycosylation

Kininogen has four *N*-linked glycosylation sites at Asn48, Asn169, Asn205, Asn294 (all of which remain on the kininogen-1 heavy chain after cleavage) and eight *O*-linked glycans at sites Thr401, Thr533, Thr542, Thr546, Thr557, Thr571, Ser577 and Thr628 [59, 60, 62, 70, 139]. While no overall or site-specific glycosylation analysis has been performed yet, core-fucosylation has been reported for sites Asn48, Asn205 and Asn294 on the basis of RP LC-MS^n^ after capturing with *L. culinaris* lectin [67]. In addition, two-dimensional gel electrophoresis, with staining for triantennary structures carrying sialyl-Lewis X has demonstrated the natural presence of this epitope on the protein, as well as its upregulation in patients with stomach cancer [140]. Kininogen is expected to be highly glycosylated by large *N*- and *O*-glycan structures, as the observed protein mass is more than 40 kDa higher than the mass calculated from the amino acid sequence. Kininogen glycosylation changes due to diseases have not yet been described.

## Serotransferrin (P02787)

Serotransferrin (STF), also known as transferrin, β1 metal binding globulin or siderophilin, is a 698 amino acid protein (19 amino acids of which are signal peptide) with a molecular mass of approximately 77 kDa (without glycosylation) [8, 337]. The protein consists of two globular domains, the N-lobe and the C-lobe which divided into two subdomains each (N1, N2, C1 and C2). The two main domains are connected by a short linker peptide [337–339]. The N-lobe is 336 amino acids in size and spans from Val25 to Glu347, while the C-lobe is 343 amino acids long and ranges from Val361 to Lys683 [337]. The lobes can interact to form a hydrophilic metal ion binding site [337]. STF is mostly produced by hepatocytes, although other tissues have also shown expression, albeit at significantly lower amounts [337]. The plasma concentration is highly stable from the age of 2 years on, with a range between 2 and 3 mg/mL [337, 340]. Levels may increase during pregnancy up to 5 mg/mL [141].

STF is an iron binding protein and it regulates iron levels in biological fluids. It can bind two Fe^3+^ ions and transport those throughout the body, avoiding the toxicity of free radical formation that may be caused by free Fe^3+^ ions [337]. Iron is essential for DNA replication as it is a co-factor of ribonucleotide reductase [341]. Several studies have shown that the number of transferrin receptors at the surface of cells was closely correlated with their proliferation state and their iron status [142, 342]. In addition, STF has been associated with several diseases like atransferrinemia and cardiovascular diseases [337]. In inflammation and allergic reactions, the STF levels are found to be significantly reduced in plasma [337]. The protein has also shown potential as a therapeutic agent. For instance, oxidative damage caused by radiotherapy can be reduced by infusion with apo-transferrin [343]. The proprieties of STF and its receptor can be exploited to deliver drugs specifically into the brain and cancer cells [344]. Additionally, conjugates consisting of the protein and a drug have been shown to yield high specific cytotoxicity (*e.g.* Tf-ADR *versus* HeLa, HL-60 and H-MESO-1 cell lines) [344, 345].

### Glycosylation

STF has two *N*-glycosylation sites located at Asn432 and Asn630 and a potential minor site at Asn491 (Asn-X-Cys) [8, 59, 141, 143, 144]. Around 6 % of the total weight of the protein is due to the carbohydrate content [142]. Lectin mobility (ConA – Sepharose column) followed by sequential exoglycosidase treatments on two STF samples of healthy patients showed that, overall, the main glycans are A2G2S2 (96–97 %), FA2G2S2 (2–3 %) and A3G3S2 (1 %) [145]. The glycosylation per site has been studied using nano-LC-ESI-MS combined with exoglycosidase treatment [143, 144]. The sites at Asn432 and Asn630 proved to be the main contributors to the total glycome, while the non-standard glycosylation site at Asn491 was glycosylated at a level of approximately 2 % [143, 144].

The glycans present on Asn432 are A2G2S2 (93.5 %), A3G3S2 (2.5 %), A2G2S1 (2.4 %) and A2FG2S2 (1.6 %), while Asn630 contains A2G2S2 (85.9 %), FA2G2S2 (6.9 %), A2FG2S2 (2.8 %), A2G2S1 (2.2 %), A3G3S2 (1.0 %), as well as some lower abundant species with increased fucosylation [143, 144]. The fucosylated antenna is most likely of sialyl-Lewis X type (at the α1-3-linked arm, β1-4-linked antenna) as it was shown by NMR spectroscopy of material purified from the amniotic fluid of pregnant women [146]. A single type of glycosylation was detected on the minor glycosylation site at Asn491, namely A2G2S2 [143]. STF *N*-glycosylation has been investigated in other biologic fluids like CSF, where similar structures have been found along with some disease-related ones [8, 147].

The glycosylation of STF has shown to be different across fluids and phenotypes with the abundance of A2G2S2 being significantly reduced in human amniotic fluid (55 %) or in the plasma of hepatoma patients (37–63 %) [145, 146]. The percentage of triantennary structures is largely increased in amniotic fluid to 32 %, while the abundance of triantennary structures in the serum of hepatoma patients ranges from 21 to 63 % [145, 146]. Abnormal isoforms of serotransferrin, especially variation in the sialic acid content, are a very sensitive and reliable biomarkers of many CDGs, and potentially for idiopathic normal pressure hydrocephalus (iNPH) patients [147, 148]. Isoelectric focusing (IEF) of serotransferrin is the first test used to rapidly reveal *N*-glycosylation related CDGs while apolipoprotein C-III is the protein of choice for the test of *O*-glycosylation related CDGs [149, 150]. Interestingly, carbohydrate deficient STF levels can also be used as indicator of heavy alcohol usage, even post mortem [8, 151].

## Vitronectin (P04004)

Vitronectin (VN), also called S-protein, serum spreading factor, or V75, is a 459 amino acid 52.4 kDa member of the pexin family and of the adhesive glycoproteins group [346–349]. The apparent molecular mass of 75 kDa is due to post translational modifications including glycosylation. VN is mainly produced in the liver and it is found in plasma at concentration of 0.2–0.4 mg/mL, where it is mostly present in monomeric or dimeric form [34, 348]. VN is also found in other body fluids such as seminal plasma, urine, amniotic fluid, CSF, bronchoalveolar lavage fluid and in platelets [348].

VN is an adhesive glycoprotein, and shows a role in blood coagulation, extracellular matrix binding, regulation of cell adhesion and spreading, and innate immunity [346, 347]. It also protects the membrane from the damages caused by the terminal cytolytic complement pathway. Underexpression of the protein has been correlated with liver conditions like fibrosis, while elevated levels have been reported in inflammatory states [350–352]. VN is also found to be implicated in HCC where specific glycoforms have been identified [152].

### Glycosylation

Three *N*-glycosylation sites have been identified in VN at Asn86, Asn169 and Asn242 by LC-MS(/MS) [59, 70]. Without site specificity, the major VN carbohydrate forms reported by LC-fluorescence are diantennary and triantennary complex type glycans, with a low percentage of hybrid structures [153]. Sialic acids are mainly found α2-6-linked, as determined by sialidase and acid treatments, followed by NMR. About 19 % α2-3-linkage has been detected on the α1-6-arm of the diantennary structures and on the β1-6-linked *N*-acetyllactosamine of the α1-3-arm of triantennary structures. Core fucosylation of vitronectin accounts for 7.9 % [153].

When looking at the glycosylation in a site-specific manner by trypsin digestion and LC-ESI-MS(/MS) analysis, Asn86 shows mainly A2G2S2 species (45 %), as well as A3G3S3 (33 %) and A3FG3S3 (20 %) [152]. At Asn169, a higher variety of glycan structures are observed. Next to the fully sialylated diantennary structures (A2G2S2, 76 %) and its monosialylated variant (6 %), around 18 % sialylated hybrid structures have been detected (ranging from 3 to 5 mannoses). Asn242 bears diantennary di- and monosialylated *N*-glycans (A2G2S2, 50 %; A2G2S1, 20 %), with possible core fucose on the fully sialylated variant (FA2G2S2, 10 %). In addition, triantennary fully sialylated structures have been detected with and without fucose (A3G3S3, 10 %; FA3G3S3, 10 %) [152].

Core fucosylation of VN has been reported at Asn242 in healthy individuals and on Asn86 in HCC patients [67]. Hybrid type and fucosylated glycans of VN have been reported to increase in patients suffering from HCC and other cancers, and thus shows potential as biomarker [152, 154]. A possible explanation for the increase of hybrid type glycans is that the alpha mannosidase in the Golgi apparatus is suppressed in HCC [17].

## Zinc-alpha-2-glycoprotein (P25311)

Zinc-alpha-2-glycoprotein (ZAG, not to be confused with *ZAG* which is the short name of its *AZGP1* gene), also abbreviated Zn-alpha-2-glycoprotein or Zn-alpha-2-GP, is a 41 kDa glycoprotein (15 % of the mass being carbohydrate) comprising a single 298 amino acid chain (20 amino acid signal peptide), with two intra-chain disulfide bridges [155, 353, 354]. The protein is produced by the liver and occurs in plasma at concentrations around 0.03–0.11 mg/mL with a mean at 0.05 mg/mL. As with many plasma glycoproteins, the functions of ZAG are diverse. The protein has been shown to interact with the beta-3-adrenoreceptor on adipocyte cells, inducing the depletion of fatty acids [355]. While its serum variant originates from hepatocytes, ZAG is expressed in many cell types including adipose tissue, buccal cells and prostate epithelial cells, and occurs in many body fluids like seminal fluid where its concentration is six time higher than in serum [355]. Functions of the on-site produced ZAG include fertilization, melanin production, regulation of the immune response, and many others. In addition, the serum concentration of ZAG shows a large increase in various types of cancer, making it a particularly good biomarker for female breast and male prostatic carcinomas [355].

### Glycosylation

Four *N*-glycosylation sites have been detected on ZAG at Asn109, Asn112, Asn128 and Asn259 [59, 60, 70, 129, 156, 157]. For three of the sites (Asn112, Asn128 and Asn259) proton nuclear magnetic resonance (^1^H-NMR) spectroscopy has revealed the major *N*-glycan structure to be diantennary and disialylated A2(2)G2(4)S2(6) [155]. For Asn259 specifically, partial sialylation (90 %) of the α1-6-linked antenna has been reported. The general presence of diantennary *N*-glycans, and the sialylation thereof, has been verified by proteomic experiments, but to date no extensive study has been made on its glycan microheterogeneity [96, 158]. Asn109 and Asn128 have for instance been suggested to carry in part core fucosylated N-glycans, but this has hitherto remained unconfirmed [67]. No information about the effect of diseases on ZAG glycosylation was found in literature.

## Immunoglobulins

Immunoglobulins (Igs) are a major component of the adaptive immune system [159]. There are five distinct classes in humans (IgA, IgD, IgE, IgG and IgM), which all share common components. Generally, immunoglobulins consist of two heavy chains and two light chains. These chains contain one variable part (H_L_ or V_L_, respectively) and three or more constant domains on the heavy chain (C_H_*n*), or one on the light chain (C_L_). Furthermore, immunoglobulins can be subdivided into a fragment antigen-binding (Fab) and a fragment crystallizable (Fc) portion. The Fab domain consists of the V_H_ and V_L_ domains and the adjacent *N*-terminal constant C_H_1/C_L_ domain. The Fc domain is built up of the remainder of the heavy chains. Immunoglobulins thus contain two Fab domains per Fc domain. Each immunoglobulin has its own specific heavy chains (α, δ, ε, γ or μ) which are joined by one or more disulfide bridges. The light chain can occur in two variants (λ and κ) that are shared by all immunoglobulins. Some immunoglobulins additionally contain a flexible hinge region between the C_H_1 and C_H_2 domains (IgA, IgD and IgG). The remaining immunoglobulins (IgE and IgM) have a rigid Ig domain instead of a hinge region. Immunoglobulin *N*-linked glycosylation occurs mostly on the heavy chains, accounting for between 2 and 14 % of the protein weight. However, the light chain can also contain *N*- and *O*-linked glycans [159].

## Immunoglobulin A (P01876; P01877)

Immunoglobulin alpha (IgA) is an antibody that exists in two subclasses (IgA1 and IgA2), and in both mono- and dimeric form. Compared to IgA2, IgA1 contains a 13 amino acid extended hinge region, which is heavily *O*-glycosylated [356, 357]. Serum IgA consists mostly of the 160 kDa IgA monomer (mIgA), has a concentration of 2.62 mg/mL (of which approximately 90 % is IgA1), and is produced by the bone marrow [357, 358]. Secretory IgA (sIgA) is observed at mucosal surfaces and produced locally, mainly occurring as a dimer of two mIgA units and a set of two connecting peptides, the J-chain and the secretory component [356, 358]. Secretory IgA is a key player in the immune defense at mucosal surfaces. Pathogenic microorganisms are prevented from attaching to the mucosal surface by sIgA surrounding the pathogen, which is then repelled by the mucosal surface due to the high abundance of hydrophilic amino acids and glycosylation [356]. The precise role of IgA in the circulation is not clear.

### Glycosylation

IgA is *N*-glycosylated at Asn144 (IgA1) and Asn131 (IgA2) in the C_H_2 domain as well as at Asn340 (IgA1) and Asn327 (IgA2) and in the tail piece domain [160]. IgA2 contains two additional *N*-glycosylation sites at Asn47 of the C_H_1 domain and at Asn205 of the C_H_2 domain [160]. While glycosylation studies have been performed for IgA1, the glycosylation of IgA2 remains to be characterized.

The main *N*-glycans present on IgA1 as detected by hydrophilic interaction liquid chromatography (HILIC)-HPLC with exoglycosidase digestion are diantennary disialylated (A2G2S2, 24 %), diantennary monosialylated (A2G2S1, 20 %) and fucosylated diantennary bisected disialylated (FA2BG2S2, 14 %) [161]. The amount of nonsialylated glycans detected was marginal, being at levels for total IgA of 6 % for the Fc region and 2 % for the Fab region [161]. A site-specific study showed that the fucosylated glycans are mostly present on the Asn340 site [162]. Furthermore it was shown that the main glycoform on the IgA1 Asn144 containing glycopeptide was A2G2S1, while the Asn340 containing glycopeptide carried mainly the FA2G2S2 [163].

The role of *N*-glycosylation of IgA in diseases is not well understood. The glycan might have an influence on the binding of IgA to the FcαR receptor, although this finding was not validated in a later study [161, 164]. Binding to the FcαR receptor can induce a pro- or anti-inflammatory response [165]. In addition, the presence or absence of sialic acids on the glycans may influence the clearance of IgA from the circulation by the asialoglycoprotein receptor [159].

## Immunoglobulin D (P01880)

Immunoglobulin delta (IgD) has, compared to the other immunoglobulins, a rather long hinge region of 64 amino acids resulting in a total apparent mass of 175 kDa. The average concentration of IgD in plasma is 0.03 mg/mL, but it can range from <0.003 to 0.4 mg/mL without any clear sex or age dependence [359–361]. Compared to the other immunoglobulins, it has a short half-life of 2.8 days [362]. IgD can be found in a secreted isoform (sIgD), as well as membrane bound on immature B cells [363]. The protein is involved in immunity and inflammation, by binding to respiratory bacteria, resulting in clearance [32].

### Glycosylation

Three *N*-glycosylation sites have been identified on the heavy chain of IgD, located at Asn225, Asn316 and Asn367, as well as seven *O*-glycosylation sites at Ser109, Ser110, Thr113, Thr126, Thr127, Thr131 and Thr132 [166–168]. HILIC-HPLC analysis with exoglycosidase digestion on released IgD *N*-glycans revealed a mixture of high mannose and complex type glycosylation. The total pool contained 35 % core fucosylation, <1 % terminal *N*-acetylglucosamine, 33 % bisection, 20 % terminal galactosylation and 31.5 % monosialylation and 21.5 % disialylation (sialic acids being α2-6-linked). The most abundant glycoforms detected were Man8 (14.4 %), Man9 (13.5 %), FA2G2S2 (7.6 %), FA2G2S1 (7.3 %), FA2BG2S2 (6.5 %) and A2G2S1 (6.1 %). Interestingly, monoglucosylated Man8 and Man9 (Man8Glc, Man9Glc; 2.4 %, 3.3 %) were observed as well [169]. The high mannose glycans were preferably found at the Asn225 site, while the complex type glycans were abundant at Asn316 and Asn367 [168]. The absence of glycans at Asn225 has been shown to completely inhibit the secretion of IgD [170].

## Immunoglobulin E (P01854)

Immunoglobulin epsilon (IgE) is a 188 kDa antibody lacking a hinge region, but that instead contains an extra C domain in its heavy chain [364]. The protein exists as a membrane-bound receptor form and in a soluble form [365]. IgE has a serum concentration of around 0.3 μg/mL, making it the lowest abundant immunoglobulin [366]. No in-depth study has yet been performed to elucidate where IgE is synthesized [364]. The primary function of IgE is the induction of an anti-parasitic immune response by activation of mast cells and basophils through the FcɛRI receptor [367]. This mechanism is also proposed to play a role in the formation of allergic responses [366, 368].

### Glycosylation

Carbohydrates form 12 % of the IgE molecular mass, making it the most glycosylated antibody in plasma [171]. The protein contains six *N*-glycosylation sites, located at Asn21, Asn49, Asn99, Asn146, Asn252 and Asn275 [172]. An additional potential site at Asn264 was not found to be glycosylated [173]. HILIC-HPLC with exoglycosidase digestion of 2-aminobenzamide-labeled glycans showed the overall species to be mainly core-fucosylated diantennary with either one sialic acid (FA2G2S1) or two (FA2G2S2) (18 and 25 % respectively) [169]. These glycans have, to lesser extent, been found in non-fucosylated form (A2G2S1, 11 %; A2G2S2, 11 %), and with bisecting *N*-acetylglucosamine (FA2BG2S1, 8.2 %). In addition, 14.2 % of the glycan pool proved to be high-mannose type (mainly Man5) [169].

By performing site-specific analysis by LC-MS/MS of tryptic glycopeptides, it was found that Asn21 mainly contains FA2G2S1 (30 %), FA2BG2S1 (30 %), FA2G2S2 (15 %) and FA2BG2S2 (10 %), while Asn49 is occupied by FA2G2S2 (30 %), FA2G2S1 (18 %), FA2BG2S2 (15 %), and FA2BG2S1 (15 %) [173]. Asn99 contains FA2G2S2 (40 %), FA2G2S1 (20 %) and bisected species in lower relative abundance (<10 %). Asn146 is glycosylated by FA2G2S2 (50 %), FA2BG2S2 (30 %), and around 10 % of FA2G2S1, while Asn252 is highly bisected, mainly containing FA2BG2S1 (35 %) and FA2BG2S2 (25 %), as well as a lesser amount of the nonbisected species FA2G2S2 (15 %) and FA2G2S1 (10 %) [173]. Interestingly, the Asn275 site almost exclusively shows oligomannosidic structures, the main species being Man5 (50 %), but higher numbers of mannoses are found as well (Man6, 15 %; Man7, 10 %; Man8, 10 %; Man9, 5 %) [171, 173].

In the same study, IgE from hyperimmune donors was seen to be similarly glycosylated as normal IgE, while IgE derived from monoclonal myelomas showed the loss of bisection and a drastic appearance of triantennary structures (up to 50 % per site) [173]. There is evidence that IgE glycosylation is important in binding to the FcϵRI receptor and can be implicated in the initiation of anaphylaxis [174, 175]. In contrast, other sources indicate that the glycans in the Fc region are only minor contributors to the binding of IgE to the FcϵRI receptor [176].

## Immunoglobulin G (P01857; P01859; P01860; P01861)

Immunoglobulin gamma (IgG) is a glycoprotein with a total molecular mass of approximately 150 kDa [159, 177]. The light chain consists of a domain covering the variable region (V_L_) as well as a constant (C_L_) domain. The heavy chain contains four domains with one domain which comprises the variable region (H_L_) followed by three constant domains: C_H_1, C_H_2 and C_H_3. Each light chain is paired with the H_L_ and C_H_1 domain of the heavy chain to form a Fab portion, whilst C_H_2 and C_H_3 domains of the two heavy chains together form the Fc portion. Between the two Fab portions and the Fc portion a flexible hinge region is positioned, which makes it possible for the two Fab arms to move individually [159, 364]. The antibody is highly stable with a half-life of approximately 12 days [369, 370]. Based on the amino acid sequence of the constant regions of the heavy chains, IgGs can be divided into four subclasses namely, IgG1 (P01857), IgG2 (P01859), IgG3 (P01860) and IgG4 (P01861) [371, 372]. Notably, IgG3 has a larger hinge region (62 amino acids) compared to the other subclasses (12 amino acids).

During a secondary immune response IgG is secreted in high amounts by B cells [373]. In healthy individuals the concentration of IgG in serum is between 7 and 18 mg/mL [374]. The average subclass-specific concentrations in plasma are reported as 5.03 mg/mL for IgG1, 3.42 mg/mL for IgG2, 0.58 mg/mL for IgG3 and 0.38 mg/mL for IgG4 [375]. IgG molecules are important for activating the complement system through the classical pathway (antibody-triggered) as well as binding to specific receptors on macrophages and neutrophils [364, 373]. The IgG subclasses differ in their ability to activate the complement system. The primary activators of the complement system are IgG1 and IgG3, whereas IgG2 can also activate it at a lower level. IgG4, on the other hand, is not capable of activating the complement system [364]. Furthermore, IgG molecules are the only antibodies that can pass from a mother to her child via the placenta, and maternal IgG has been shown to gradually decrease throughout pregnancy [364, 376, 377].

The majority of therapeutic antibodies is derived from IgG1, where the glycosylation plays an important role for their function [35, 364, 377–380].

### Glycosylation

Glycosylation can occur on both the Fc and Fab portions of the IgG molecules [178]. The Fc region has been extensively studied with a highly conserved *N*-glycosylation site in the C_H_2 domain at Asn297. Notably, this site may have a different number for different IgG subclasses and variants [178].

Another possible *N*-glycosylation site may be found at Asn322 of IgG3 although no occupation has yet been described [178]. The Fab portion is known to be *N*-glycosylated in 15–25 % of the cases [179].

The overall glycosylation of IgG has been the subject of many studies using a variety of different methods [24]. In a recent glycosylation MALDI-TOF-MS study on a released glycan level, the most abundant glycans were complex types, *i.e.* FA2G1 (31 %), FA2G2 (23 %), FA2G2S1(6) (13 %), FA2 (10 %) and FA2BG1 (5 %) [180]. Only a small portion of the glycans were found to be high mannose (0.21 %), of which Man8 (0.06 %) was the most abundant, followed by Man9 (0.05 %). Overall, 92 % of the total IgG pool was core-fucosylated, 13 % bisected, 18 % monosialylated and 3 % disialylated. Twelve percent of the glycans contained α2-6-linked sialic acids, against 0.2 % α2-3-sialylated species [180]. These findings are in agreement with previously reported sialylation values obtained by lectin interaction [181]. Next to the study of overall IgG glycosylation, differences between the Fab and Fc have also been studied by MALDI-TOF-MS after affinity capturing of the different regions [180]. The Fc region shows a similar profile as the total IgG profile, albeit with a lesser degree of sialylation. FA2G1 (32 %) was again the most pronounced glycan, followed by FA2G2 (27 %), FA2G2S1(6) (15 %), FA2 (9 %) and FA2BG1 (5 %), while the amount of high mannose type species was found to be very low (0.1 %). In contrast to Fc, the Fab region showed a significantly higher degree of sialylation, with 40 % of the species being monosialylated, and 52 % being disialylated. Also bisection and high mannose species were seen to be higher (45 and 4 % respectively). Specific compositions included FA2BG2S1(6) as most abundant with 21 %, followed by A2G2S2(6) (17 %), FA2BG2S2(6) (16 %), FA2G2S2(6) (16 %) and FA2G2S1(6) (10 %). For the high mannose types Man6 was the most abundant with 1.2 % followed by Man8 (1.0 %) and M5 (0.7 %) [180].

Affinity capturing followed by LC-MS with CID and electron-transfer dissociation (ETD) fragmentation of tryptic IgG glycopeptides revealed that the various IgG subclasses are similarly glycosylated, but with some notable differences [182, 183]. IgG1 tends to show higher galactosylation than the other subclasses, whereas IgG2 shows the highest degree of core-fucosylation and IgG3 the least. IgG4 was found more difficult to study due to its relatively low abundance [182]. Another study examined the *O*-glycosylation of IgG3 in the hinge region, revealing that the threonine sites (T) in the three repeated peptide sequences (CPRCPEPKSCD**T**PPP) are partially occupied with core 1-type *O*-glycans [184].

A vast body of literature exists describing disease-associated changes of IgG glycosylation, as well as the regulation and immunological effects of such glycosylation changes. In the following, only a very concise view of this field will be given, and we would like to refer the interested reader to more specialized reviews [177, 185, 186].

IgG glycosylation has been strongly associated with age, with a negative correlation between age and galactosylation [19, 20, 187, 188]. IgG FA2 seems to have a strong pro-inflammatory effect through various mechanisms, *e.g.* the lectin pathway of the complement system [19, 188, 189]. Increased levels of FA2 glycans and/or lowered levels of FA2G2 glycans is found in many diseases, including rheumatoid arthritis, Crohn’s disease, granulomatosis with polyangiitis, tuberculosis, HIV and myositis [189–193]. Several studies revealed that core-fucosylation is an important factor in the binding capacity of the Fc region to the Fc_ϒ_RIIIa receptor [194, 195]. The lack of core-fucosylation suggests improvement in the binding extensively resulting in a higher degree of antibody-dependent cell mediated cytotoxicity (ADCC) receptor [194, 195]. The presence of sialic acids is able to reduce the binding capacity of the antibody to the Fc_ϒ_RIIIa receptor, as a consequence the activity of ADCC is decreased and anti-inflammatory effects are enhanced, although this only appears to be the case for α2-6-linked sialylation [33, 178, 196, 197]. Interestingly, during pregnancy the glycosylation also appears to change, especially in the Fc region where the levels of galactosylation and sialylation increase [28, 198–200] . This might be to suppress the immune response of the mother against her child [198]. Alterations in glycosylation have also been reported to occur in a subclass specific level, for example in patient suffering from hepatocellular carcinoma, cirrhosis, or myositis [190].

## Immunoglobulin M (P01871)

Immunoglobulin mu (IgM) is a 970 kDa (in pentameric form) antibody, consisting of five 190 kDa subunits (of 452 amino acids) [159, 364, 381]. The protein can be membrane-bound on the B1- and B2-cells where they are produced [382], or secreted into blood at plasma concentrations of 0.5–2.5 mg/mL [374]. In circulation, IgM exists either as a pentamer with coupling J-chain, or occasionally as hexamer lacking the J-chain [383]. No hinge region has been reported for the immunoglobulin, but it contains an extra C domain instead [364]. In total, pentameric IgM consists of 10 heavy chains, 10 light chains and 1 J-chain, arranged in a mushroom-shaped molecule, leading up to being the largest antibody in human plasma by far [384].

IgM antibodies are an early and main activator of the classical complement pathway [364], but also play a role in homeostasis, inflammation, infection, atherosclerosis and autoimmunity [381]. The protein is additionally involved in apoptosis [381], as absence of IgM shows a three- to four-fold decrease in apoptotic cell uptake by macrophages [385]. Furthermore, IgM has been described in several studies regarding acute coronary syndromes and cardiovascular diseases, where an elevated urine excretion of IgM has been reported [386, 387]. The presence of glycans on non-antigen bound IgM may also assist the agglutination of virus particles present in serum via viral lectin hemagglutinins [159].

### Glycosylation

IgM contains *N*-glycosylation sites at Asn46, Asn209, Asn272, Asn279 and Asn439, of which Asn439 is only 17 % occupied [159, 201, 202]. HILIC-HPLC-MS has shown the overall *N*-glycosylation to be mainly diantennary with core fucosylation, either with- or without bisecting *N*-acetylglucosamine (FA2BG2S1, 26 %; FA2G2S1, 19 %), followed by the oligomannose compositions Man6 and Man5 (10 and 6 %, respectively) [202]. By lectin capturing, the high-mannose compositions have been attributed to Asn297 and Asn439, leaving the complex type glycosylation to be found at Asn209 and Asn272 [159, 203]. Interestingly, the partially occupied Asn439 shows larger high-mannose structures (Man6-8) than site Asn297 (Man5-6), suggesting that the former is difficult to access by both the dolichol precursor and the mannosidases required for trimming of the structure. Depending on the source, Asn46 can be occupied by either oligomannose or complex type *N*-glycans [159, 203].

## Discussion

Here we present an overview of the *N*-glycosylation of 24 major plasma glycoproteins. It has been shown for many of these proteins that glycosylation changes are implicated in serious pathological states such as cancers, autoimmune diseases and CDG and that their glycosylation pattern could be used as biomarkers, prognostic tools or even as anchor points for targeted treatments [15, 35, 148, 388, 389]. These findings have resulted in an increasing interest in the glycosylation analysis of easily accessible biofluids such as plasma, and many correlations have been established between disease (states) and the abundance of specific glycosylation traits. Recent advances in sample preparation methods, separation techniques, mass spectrometry, and the development of robotized platforms are easing routine analysis of the total plasma *N*-glycome and allow the screening of large cohorts in reasonable times, thus enhancing the possibilities to search for putative biomarkers and predictions tools [38, 109, 390–392]. This, however, requires that the observed differences in glycosylation can be interpreted in a biologically meaningful manner, and traced back to changes in the levels of glycosylated proteins, and to possible glycosylation changes of specific proteins.

The information contained in this review stems from many sources and methodologies. When exploring protein glycosylation to its full complexity, this comprises the occupancy per site and the relative abundances of glycoforms present per site, as well as information on linkages and isomer distribution. No single analysis method is capable of providing all this information in a comprehensive manner, let alone on a complex sample such as human plasma. Consequently, different levels of detail are available with respect to the glycosylation of the major glycoproteins that make up human plasma. Analysis methods encountered in this review are very diverse, including NMR, lectin capturing, LC-fluorescence, as well as several mass spectrometric methods [72, 390, 393]. NMR has proven definitive for providing structural features of a glycan, but is limited by sample throughput and amount of material needed, as well as by its inability to characterize multiple species present in a complex sample [390]. Mass spectrometric methods applied in bottom-up studies of glycopeptides generally only provide glycosylation information on a compositional level, but may in addition provide site-specific glycosylation information by revealing the amino acid sequence as well as glycan attachment site [182]. As such, high-throughput mass spectrometric screening methods have been used to identify and confirm many of the glycosylation sites in human plasma, although the use of deglycosylated peptides has prevented characterization of the glycans themselves [59, 60, 67]. A particularly successful combination of methodologies for in-depth study of glycosylation has proven to be LC-MS(/MS) with exoglycosidase digestion and/or lectin capturing, which has been used to study a fair number of the proteins covered in this review [110, 169].

Well-studied proteins covered in this review include alpha-1-acid glycoprotein, alpha-1-antitrypsin, haptoglobin, serotransferrin, vitronectin, and IgG, while others such as histidine-rich glycoprotein and kininogen-1 still remain to be studied at the most basic level (Table 1). Overall characterization reveals a high degree of galactosylation and sialylation across the plasma *N*-glycome, the most abundant species for most sites having full coverage of all their antennae. Next to receptor interaction and charge induction, the terminal sialic acids are known to play a large role in determining the half-life of proteins, and less than full sialylation would lead to hepatic clearance via the asialoglycoprotein receptor [394].

Specifically, the major detected glycan species are A2G2S2 and its monosialylated variant (Fig. 1, Table 1), with β1-2-linked antennary *N*-acetylglucosamines, β1-4-linked galactoses, and α2-6-linked *N*-acetylneuraminic acids [104, 155]. These are found on the majority of glycoproteins, and are particularly abundant on serotransferrin, fibrinogen, ceruloplasmin and alpha-2-macroglobulin. Potential fucosylation for these diantennary structures mostly occurs in α1-6-linkage on the core *N*-acetylglucosamine.

The more truncated *N*-glycosylation, *i.e.* lack of sialic acid termini and incomplete galactosylation, is reserved for immunoglobulin G, and to lesser extent apolipoprotein B-100 [11, 180]. Interestingly, immunoglobulin G is the only major plasma protein we have found to contain core-fucosylation as well as incomplete galactosylation/sialylation, meaning that the TPNG species FA2, FA2G1 and FA2G2 predominantly reflect the glycosylation of this protein [180]. Similarly, immunoglobulin M is the major carrier of the bisected species FA2BG2S1, with IgG and the lowly abundant immunoglobulin E contributing to the expression of this compositional glycan to a lesser extent [169, 180, 202].

The high mannose type glycosylation is also differentially distributed. Whereas the lower size oligomannose structures Man5 and Man6 are distributed across alpha-2-macroglobulin, apolipoprotein B-100 and immunoglobulin M, the larger structure Man9 mainly originating from apolipoprotein B-100 [11, 72, 202]. In addition, the high mannose structures have been reported on the Fab portion of IgG [180].

Tri- and tetraantennary structures are found in lower abundance than the diantennary structures, and have some discerning features. Whereas diantennary glycans are mainly sialylated with an α2-6-linkage, for the triantennary species on average one in three sialic acids is α2-3-linked [54]. Furthermore, potential fucosylation is predominantly α1-3-antennary, and located at the α2-3-sialylated antenna to form sialyl Lewis X, which itself is favored on the β1-4-linked N-acetylglucosamine of the α1-3-branch [54]. These fucosylated and non-fucosylated triantennary glycans are commonly expressed in minor amounts for sites that also have diantennary glycosylation (examples including alpha-1-antitrypsin and ceruloplasmin), but represent the most abundant glycosylation type for alpha-1-acid glycoprotein. Among the proteins covered in this review alpha-1-acid glycoprotein also stands out by expressing tetraantennary *N*-glycan species (also with potential sialyl-Lewis X). Other candidates likely to contain the larger tri- and tetraantennary structures are kininogen-1 and histidine-rich glycoprotein (judged by the difference between apparent and calculated mass), but this has not been confirmed yet, possibly due to the technical difficulty associated with the analysis of these glycosylations.

**Fig. 2:**
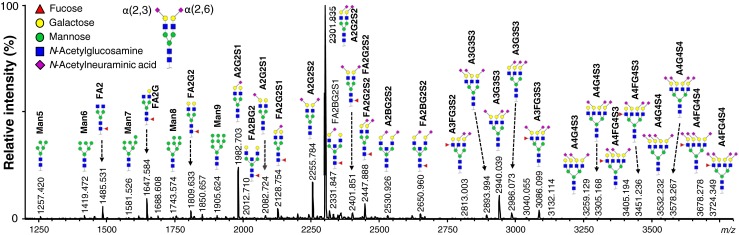
Typical reflectron positive mode MALDI-TOF-MS spectrum of the total *N*-glycosylation of pooled human plasma after enzymatic *N*-glycan release, ethyl esterification, and hydrophilic-interaction liquid chromatography (HILIC) enrichment [109]. Glycan species are assigned as [M+Na]^+^ on basis of the reviewed plasma structures. Where multiple options are possible, the most abundant has been used for assignment. Sialic acid orientation is on basis of observed mass after ethyl esterification, while the other linkages are presumed on basis of literature. For fucosylation, diantennary structures are reported to mostly carry an α1-6-linked fucose on the reducing end *N*-acetylglucosamine, while tri- and tetraantennary structures are reported to mostly have α1-3-linked antennary fucosylation in the form of Lewis X (or sialyl-Lewis X when the antenna carries an α2-3-linked sialic acid). For the tri- and tetraantennary structures, antennae representation has been simplified for readability purposes

When combining the contributions of the 24 major glycoproteins covered in this review to calculate a theoretical total plasma *N*-glycome, a remarkable congruence is observed with a TPNG profile registered for *N*-glycans released from human plasma (Figs. 1 and 2). Truncated fucosylated diantennary structures, mono- and disialylated diantennaries, as well as tri- and tetraantennary structures and their relative fucosylation are roughly in the correct ratios when compared to MALDI-TOF-MS with sialic acid-linkage-specific stabilization. The differences between the theoretical and measured *N-*glycome could be due to variations in the sample types or origins used in each study, as we have seen that phenotypes could occur at different ratios among ethnicities, but also due to the approximations in the protein concentrations that are often values averaged from multiple papers, or even to the remaining low abundant glycoproteins not covered by this review, although they should only account for a few percent of the TPNG.

In all, we expect the knowledge gathered in this review to facilitate the clinical interpretation of plasma-wide glycosylation analysis. This review underlines the necessity for further protein-specific glycosylation analysis to fill the still considerable gaps in our understanding.

## Electronic supplementary material

Below is the link to the electronic supplementary material.

Supplementary Table 1 (XLSX 38 kb)
